# A target enrichment high throughput sequencing system for characterization of BLV whole genome sequence, integration sites, clonality and host SNP

**DOI:** 10.1038/s41598-021-83909-3

**Published:** 2021-02-25

**Authors:** Nagaki Ohnuki, Tomoko Kobayashi, Misaki Matsuo, Kohei Nishikaku, Kazuya Kusama, Yasushi Torii, Yasuko Inagaki, Masatoshi Hori, Kazuhiko Imakawa, Yorifumi Satou

**Affiliations:** 1 Laboratory of Animal Health, Department of Animal Science, Faculty of Agriculture, Tokyo University of Agriculture, Atsugi, Kanagawa 243-0034 Japan; 2 Division of Genomics and Transcriptomics, Joint Research Center for Human Retrovirus Infection, Kumamoto University, Kumamoto, 860-8556 Japan; 3 International Research Center for Medical Sciences (IRCMS), Kumamoto University, Kumamoto, 860-8556 Japan; 4 Department of Endocrine Pharmacology, Tokyo University of Pharmacy and Life Sciences, Tokyo, 192-0392 Japan; 5 Department of Veterinary Pharmacology, Graduate School of Agriculture and Life Sciences, The University of Tokyo, Tokyo, 113-8657 Japan; 6 Laboratory of Molecular Reproduction, Research Institute of Agriculture, Tokai University, Kumamoto, 862-8652 Japan

**Keywords:** Virology, Retrovirus, Tumour virus infections

## Abstract

Bovine leukemia virus (BLV) is an oncogenic retrovirus which induces malignant lymphoma termed enzootic bovine leukosis (EBL) after a long incubation period. Insertion sites of the BLV proviral genome as well as the associations between disease progression and polymorphisms of the virus and host genome are not fully understood. To characterize the biological coherence between virus and host, we developed a DNA-capture-seq approach, in which DNA probes were used to efficiently enrich target sequence reads from the next-generation sequencing (NGS) library. In addition, enriched reads can also be analyzed for detection of proviral integration sites and clonal expansion of infected cells since the reads include chimeric reads of the host and proviral genomes. To validate this DNA-capture-seq approach, a persistently BLV-infected fetal lamb kidney cell line (FLK-BLV), four EBL tumor samples and four non-EBL blood samples were analyzed to identify BLV integration sites. The results showed efficient enrichment of target sequence reads and oligoclonal integrations of the BLV proviral genome in the FLK-BLV cell line. Moreover, three out of four EBL tumor samples displayed multiple integration sites of the BLV proviral genome, while one sample displayed a single integration site. In this study, we found the evidence for the first time that the integrated provirus defective at the 5′ end was present in the persistent lymphocytosis cattle. The efficient and sensitive identification of BLV variability, integration sites and clonal expansion described in this study provide support for use of this innovative tool for understanding the detailed mechanisms of BLV infection during the course of disease progression.

## Introduction

Bovine leukemia virus (BLV) is the causative agent of enzootic bovine leukosis (EBL). EBL is generally characterized by local and systemic tumor development following a long incubation period of several years after BLV infection^1–3^. It is suspected that only 5% of BLV infected cattle are thought to develop EBL. However, the number of cattle condemned at slaughterhouse with bovine leukosis, which is mostly associated with BLV, is increasing year by year in Japan^4^. The condemnation of carcasses cause severe economic losses in the cattle industry^5^.

BLV is a single-stranded RNA virus with a genome of approximately 8720 nucleotides^6^. The BLV genome is reverse transcribed to double-stranded DNA and integrated into the host genome as a provirus. The BLV proviral genome consists of four major genes coding structural proteins and enzymes, namely, Gag, Pro, Pol and Env, and at least four genes coding regulatory proteins (Tax, Rex, R3 and G4)^1^. In addition, BLV also encodes viral micro RNAs (miRNAs) and antisense RNAs (AS1 and AS2)^7,8^. Nucleotide sequences of these elements are flanked by two identical untranslated regions of 560 nucleotides, the long terminal repeats (LTR)^9^. Previous studies have demonstrated that Tax and G4 proteins are oncogenic in that these proteins facilitate transformation of primary rat embryo fibroblast^10,11^. Moreover, the G4 protein has significant roles in tumor onset and progression through the maintenance of high viral loads^12,13^. In addition to these proteins, miRNAs in the BLV genome are also reported to be oncogenic^14^. It remains uncertain how sequence diversity of the BLV genome affects pathogenicity, as well as why a minority develops EBL whereas most infected cattle remain asymptomatic. Several host single nucleotide polymorphisms (SNPs) that are associated with high viral load of infected cattle or EBL onset have been described^15–19^. However, little is known about either viral or host molecular determinants of disease progression.

Previous studies demonstrated associations of oncogenesis caused by BLV and insertional mutagenesis following proviral integration to the host genome^20,21^. During persistent infection, BLV-infected cells with proviruses inserted in nearby transcribed region are selected, and *cis*-activate the cellular transcripts by initiating transcription at the LTR promoter. The key mechanism of leukemogenesis is reported to be clonal or oligoclonal expansion of B cells. BLV-infected B cell clones selectively proliferate during the long incubation period as a result of a growth advantage possibly conferred by multiple gene mutations or epigenetic changes. However, the relationships between the BLV genome insertions, clonal expansion and disease progression in BLV-infected cattle are still unclear.

Together, the associations between disease progression and polymorphisms in each of the viral proteins and transcripts, as well as integration sites (ISs) in the host genome, and clonal proliferation of certain infected B cell clones are not fully understood. Characterization of the interactions between these factors might lead to a clearer understanding of the initiation and maintenance of leukemogenesis. Whole-genome sequencing using the next-generation sequencing (NGS) system simplifies investigation of the nature of the integrated proviral genomes and host genomes, allowing the analysis of genetic variations of the BLV proviral sequence itself and identification of BLV ISs. In addition, BLV-infected B cell clonality could be analyzed by detecting host sequences adjacent to the integrated proviral sequence. A slight disadvantage of this technique is that it requires a high sequencing depth to acquire the target BLV genome and nearby host genome from whole-genome sequencing data.

Here, we describe the application of a BLV-targeted DNA capture sequencing (DNA-capture-seq) method, enabling analysis of the integrated BLV and the host genome comprehensively. Using a panel of custom-designed and specific biotinylated DNA probes, the BLV proviral genome and the host genome sequences of interest were enriched, from which full genome sequencing of the virus was obtained. Moreover, by analyzing host sequences adjacent to enriched BLV sequences, BLV ISs were identified. Since the BLV-infected B cells maintain their chronic infection by mitotic proliferation, each clone shares the same genomic integration site. By detecting the frequency of ISs, the abundance of the clones was analyzed. Finally, the sequence data captured by host gene-specific probes were analyzed to detect host SNPs. These comprehensive genome analyses effectively promote the acquisition of the pathologically and biologically important information in disease progression or EBL progression.

## Results

### Validation of BLV capture probe

**Figure 1:**
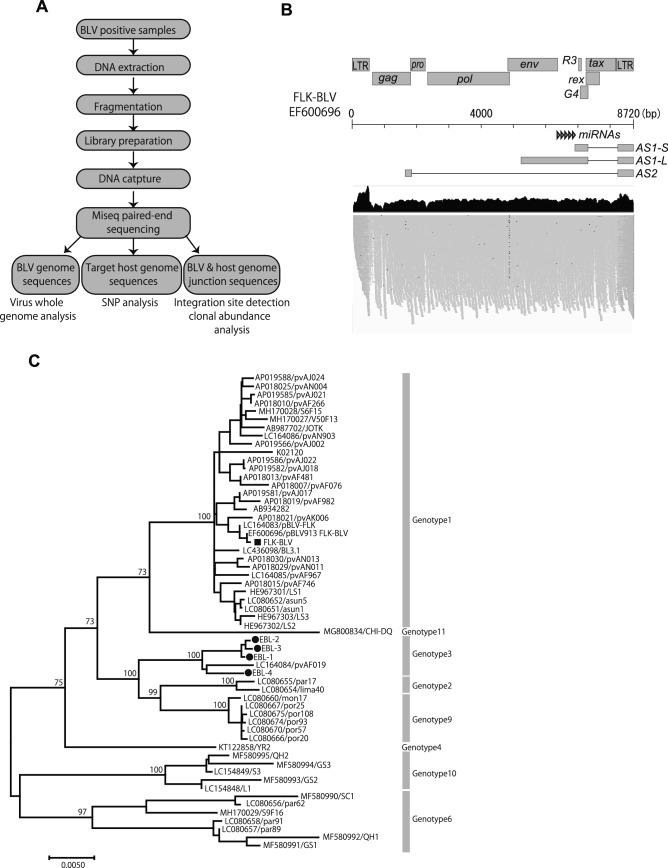
Application of DNA-capture-seq to analyze BLV proviral sequences and ISs. (**A**) Schematic diagram of the application of the target enrichment. (**B**) Visualization of sequence reads mapped to the FLK-BLV sequence (EF600696). NGS reads mapped to BLV are shown below the reference sequence. (**C**) Maximum-likelihood phylogenetic tree analysis of BLV whole genome sequences were generated with five newly obtained sequences (a sequence from FLK-BLV cell line indicated by filled square and four sequences from EBL tumor samples indicated by filled circle) together with 53 sequences from the GenBank database. The phylogenetic tree was generated and visualized using MEGA 7 with 1000 bootstrap replicates. The bar at the bottom of the figure denotes the estimated number of amino acid substitutions per site, indicating genetic variation for the length of the scale.

In order to establish a method that efficiently enriches the BLV sequences and the host genome sequences of interest, we designed custom DNA probes specific to the FLK-BLV subclone pBLV913 complete genome (EF600696)^22^ and the TNF-α promoter region of the bovine genome. Figure 1A describes the general flow chart used for enrichment of the BLV genome, the target BLV and the nearby host genome. Genomic DNA was extracted from the FLK-BLV cell line and randomly fragmented by mechanical sonication to generate DNA libraries. The resulting adapter ligated DNA libraries were hybridized to the custom biotinylated capture probes, and subsequently captured by streptavidin beads. The sample generated 0.97 million raw reads of which 0.61 million reads (63.3%) mapped to the host genome (the Ovine Genome Assembly Oar_v4.0) and 0.1 million reads (10.2%) mapped to the target BLV reference genome (EF600696). The sequence reads mapped to the target BLV reference genome covered 100% of the BLV genome (Fig. 1B). The phylogenetic analysis revealed that the full genome consensus sequence reconstructed from mapped reads was clustered in the same clade as the previously reported FLK-BLV sequence (accession numbers: LC164083 and EF600696) (Fig. 1C).

### BLV ISs in FLK-BLV cell line

**Figure 2:**
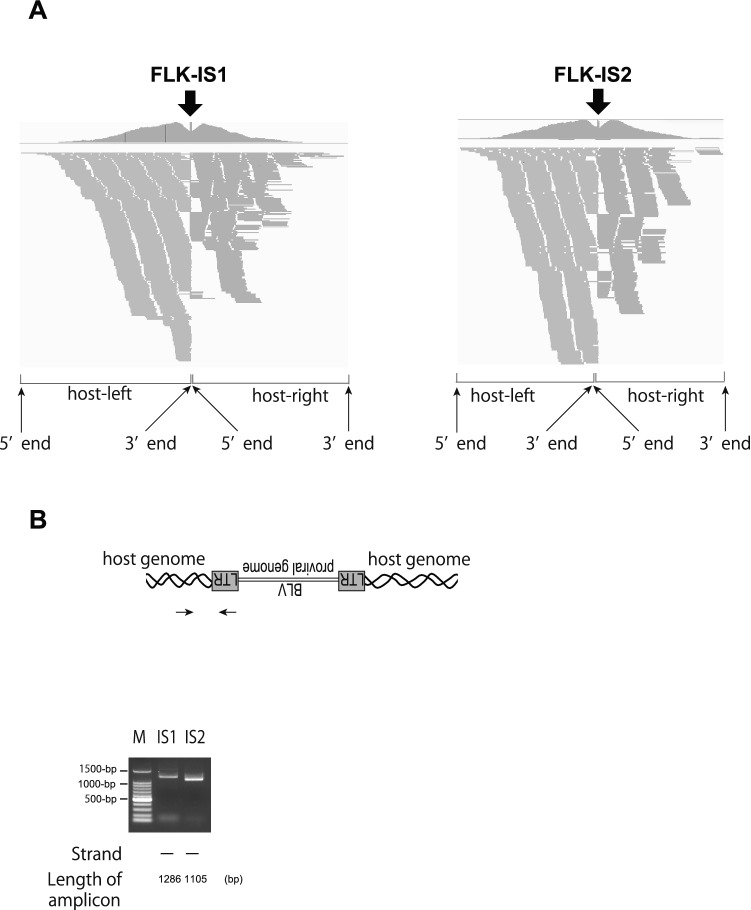
Visualization of ISs of the FLK-BLV cell line. (**A**) Visualization of NGS reads mapped to ovine genome around ISs. The arrow indicated the location of the IS. The sequences of virus-host chimeric reads next to the end of BLV gnome were indicated as host-left and host-right; respectively. (**B**) Confirmation of detected ISs by conventional PCR. *M* DNA ladder maker. Arrows indicate the positions of primers. ISs are numbered as in Table 1.

In the previous studies, FLK-BLV was often used as control of Southern blotting analysis, in which multiple copies of the BLV genomes were integrated into the ovine genome in the FLK-BLV cell line^23,24^. The BLV probes used in this study would capture virus-host chimeric DNA fragment as well as virus-only reads from the DNA library of FLK-BLV cells. We detected ISs in the FLK-BLV cell line by analyzing the host sequence of virus-host chimeric reads. BLV ISs were visualized as a hollow between two peaks of NGS reads mapped to the host genome because BLV-host junction sequences were automatically depleted by mapping algorithms (Fig. 2A). Consistent with previous studies, multiple ISs were detected (Table 1). Within these ISs, IS3 was located in the CpG island of untranslated region of Avian Myelocytomatosis Viral Oncogene Homolog (c-Myc) of the ovine genome (Table 1). To confirm these ISs, IS1 and IS2 were subjected to PCR using primers set to the host genome and BLV genome (Fig. 2B). Taken together, these results indicated that DNA-capture-seq is applicable to detect BLV ISs in the BLV-infected cell line.

**Table 1:** The list of virus-host reads detected in FLK-BLV cell line.

IS	Chromosome	Host-left			Host-right			Reads.total	Strand	Gene located within 1000bp		Within repetitive sequence
		5'end	3'end	Number of reads	5'end	3'end	Number of reads			Gene name	Intron/Exon	Region
IS1	chr1	245,437,082	245,437,600	434	245,437,586	245,437,988	355	753	–	–	–	–
IS2	chr2	9,798,618	9,799,109	761	9,799,100	9,799,546	568	1329	–	–	–	–
IS3	chr9	25,557,188	25,557,359	88	25,557,159	25,557,389	92	180	–	MYC	UTR	CpG Island

*IS* ID of each integration site, *host-left* host-right; nucleotide position of virus-host chimeric reads as shown in Fig. 2A, *number of reads* the number of virus-host chimeric reads around each IS strand, direction of BLV genome to the host genome.

### Application of DNA-capture-seq to the study of BLV infected and EBL cattle

Recent phylogenetic studies demonstrated that BLV can be classified into 11 genotypes. In Japan, two BLV genotypes, namely genotype 1 and 3, are reported. Most of BLV sequences reported in Japan are genotype 1, and only one full-length sequence of genotype 3 was reported so far (LC164084). To characterize and analyze sequence variation of BLV genotype 3, therefore the full-length BLV proviral sequences in tumor tissues of EBL cattle infected with genotype 3 were analyzed by DNA-capture-seq (Table 2). The detailed information on the cattle analyzed in this study was summarized in Table S1. The five newly assembled BLV full-length sequences were phylogenetically analyzed with 53 representative sequences from each clade. The BLV sequences from the examined tumor samples were clustered together with the BLV genotype 3 sequence (Fig. 1C). The overall nucleotide sequence identities were notably high among the obtained four sequences (estimated evolutionary *p* distance: 0.005) and between the previously reported BLV genotype 3 sequence (LC164084: estimated evolutionary *p* distance: 0.01). These sequences were aligned with FLK-BLV sequence (EF600696) and pairwise sequence identity and different nucleotides were analyzed (Fig. S1A). As shown in Fig. S1B, 42 unique amino acid substitutions observed were specific to genotype 3. Among these substitutions, 82F, 133T, and 254L were located at conformational epitope, neutralization domain 2 (ND2) and liner epitope of ENV protein, respectively. In addition, 80S was located at nuclear export signal (NES) of Rex protein. Moreover, 43K and both 164P and 185T were located at a putative zinc finger and leucine-rich activation domain of Tax protein. Although functional relevance of these substitution has not been reported so far, these substitutions may partially explain low percentage of BLV genotype 3 prevalence in Japan.

**Table 2:** The list of virus-host reads detected in tumor tissues of EBL cattle.

Sample name	IS	Chromosome	Host-left			Host-right			Reads.total	Strand	Gene located within 1000bp	Within repetitive sequence	
			5'end	3'end	Number of reads	5'end	3'end	Number of reads			Gene name	Region	Name
EBL1	IS1	chr14	4,446,728	4,447,242	2971	4,447,234	4,447,745	4113	7084	+	FAM135B	SINE	−
	IS2	chr17	35,540,227	35,540,728	966	35,540,721	35,541,235	1206	2172	+	−	LTR	ERV3-163_I-int
	IS3	chr28	41,855,125	41,855,596	929	41,855,586	41,856,050	1031	1960	+	−	LINE	−
EBL2	IS1	chr13	77,065,395	77,065,855	654	77,065,847	77,066,342	671	1325	+	LOC112449387	−	−
	IS2	chr14	70,565,534	70,565,992	1136	70,565,981	70,566,456	872	2008	−	FAM92A	−	−
EBL3	IS1	chr1	89,183,663	89,184,176	1008	89,184,165	89,184,695	1275	2283	+	−	−	−
	IS2	chr28	15,806,809	15,807,260	559	15,807,251	15,807,695	452	1011	−	ANK3	−	−
EBL4	IS1	chr17	51,625,289	51,625,860	926	51,625,851	51,626,422	1228	2154	+	LOC100847522	LINE	L2b

*IS* ID of each integration site, *host-left, host-right* nucleotide position of virus-host chimeric reads as shown in Fig. 2A, *number of reads* the number of virus-host chimeric reads around each IS, *strand* direction of BLV genome to the host genome, *FAM135B* Family With Sequence Similarity 135 Member B, *FAM92A* family with sequence similarity 92 member A, *ANK3* Ankyrin 3.

To analyze the sequence variation of BLV whole genome, variant calling between consensus sequences of analyzed samples and FLK-BLV (EF600696) were determined. Of these variants, deletions and insertions were not detected. Pairwise analysis at nucleotide level revealed an average identity of 0.97 between full length of EF600696 and BLV genotype 3 (Fig. S1A).

To test the positive selection in BLV genotype 3, the sequences of six open reading frames (ORFs) (*G4*, *gag*, *pro*, *pol*, *env* and *tax*) and *AS1* were aligned using ClustalW in MEGA7, and phylogenetic trees were reconstructed (Fig. S2 and data not shown). The six ORF sequences of obtained consensus sequences were all clustered with the previously reported genotype 3 sequence. Evidence of positive selection of the internal branches among the six ORFs was calculated by branch site test. Although the *p* values of likelihood ratio test (LRT) through the use of the Bayes Empirical Bayes (BEB) method were not statistically significant (0.16 and 1.0), we observed weak evidence of positive selection at five amino acid sites in G4 and Tax (Table 3).

**Table 3:** Branch-site analysis of the BLV genotype 3.

ORF	− 2ΔlnL	*p *value	Positive selection site by BEB (posterior probability)
*G4*	1.00	0.32	I40(0.84), K41(0.82), R60(0.83)
*gag*	0.00	1.00	ND
*env*	0.00	1.00	ND
*pro*	0.00	1.00	ND
*pol*	0.00	0.99	ND
*tax*	0.00	1.00	R43(0.51), L305 (0.69)

*ND* not detected, *BEB* Bayes Empirical Bayes.

**Figure 3:**
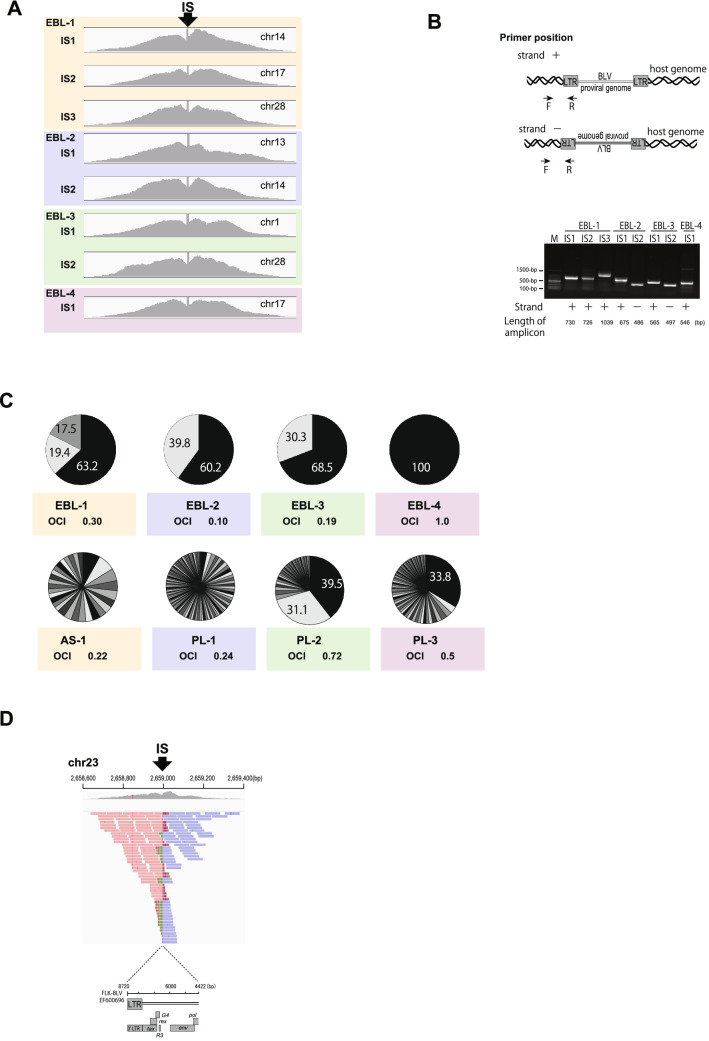
Visualization of ISs of EBL tumor, AS and PL blood samples. (**A**) Visualization of NGS reads mapped to the bovine genome around ISs. The arrow indicates the integration breakpoints in the bovine genome. (**B**) The pie-charts depict the distribution of clonal abundance of each EBL tumor sample, AS and PL blood sample. Each slice represents a BLV-infected clone and the size of the slice represents the relative abundance of the clone and the number indicates the proportion (%) of each clone. Oligoclonality index (OCI) measures the non-uniformity of the clonal abundance. ISs are numbered as in Table 2. (**D**) Visualization of NGS reads mapped to the bovine genome around the defective provirus of PL-2. The arrow indicates the integration breakpoints in the bovine genome.

We then assessed ISs and proportions of each clonal population through detailed analysis of the host sequences adjacent to the BLV proviral genome (Fig. 3, Table 2). Among four tumor samples, a single IS was detected in one sample (EBL-4) (Fig. 3A), indicating that this EBL cattle tumors derived from a single BLV-infected cell with monoclonal expansion. In contrast to previous studies which reported that most of the EBL cattle have BLV-infected cells with single IS^25,26^, multiple ISs were detected (EBL-1, EBL-2 and EBL-3). To confirm these ISs, PCR using primers set to the host genome and the BLV genome were performed (Fig. 3B). Host sequences were not detected adjacent to the internal sequence of BLV provirus, indicating that the 3′ end and 5′ end of detected proviruses were conserved in all tumor samples (data not shown). Previous studies have demonstrated that BLV ISs in EBL cattle are often found in retroelements: Short Interspersed Nuclear Element (SINE), Long Interspersed Nuclear Element (LINE) and Long terminal repeat (LTR) of endogenous retrovirus^25^. In our current study, four out of eight ISs were detected in retroelements, possibly resulting from negative selection of clones integrated into transcriptionally inactive region as previously reported^20^ (Table 2). ISs were also located within introns of Refseq genes: Family with sequence similarity 92 member A (FAM92A) that acts as a positive regulator of ciliary hedgehog signaling, Ankyrin 3 (ANK3) which is the structural constituent of cytoskeleton and uncharacterized gene LOC100847522), although the functional relevance of these genes to EBL are not known.

To measure the dispersion of abundance of each BLV-infected cell clone, the oligoclonality index (OCI) was calculated based on the Gini coefficient as previously described^20^. OCI ranges from 0 to 1, with 0 indicating that all the BLV-infected clones have the same abundance and 1 corresponding to a complete monoclonal population. Three out of four tumor samples, OCI is relatively low (0.30 for EBL-1, 0.10 for EBL-2 and 0.19 for EBL-3, indicating that these multiple clones are abundant in substantial proportions rather than composed with major population with minor populations.

Taken together, these results indicated that DNA-capture-seq is applicable to analyze the BLV whole genome sequences, ISs as well as clonal expansion of infected cells in the tumor tissues of EBL cattle infected with BLV genotype 3.

### Application of DNA-capture-seq to the study of asymptomatic (AS) and persistent lymphocytosis (PL) cattle

Blood samples from AS and PL cattle infected with BLV genotype 1 were analyzed to confirm the further application to the different stages and the different genotype. The variant calling between consensus sequences of analyzed samples and FLK-BLV (EF600696) were demonstrated (Fig. S1A). Of these consensus sequences, deletions and insertions were not detected.

Most of integration breakpoints of ISs of AS, PL-1 and PL-3 samples were mapped next to the 5′ and 3′ end of LTR, indicating that full length proviral genomes were conserved in these samples. There were two remarkably dominant proviruses in PL-2 (Fig. 3C). One (IS1) was mapped next to the 3′ LTR and internal sequence of the BLV genome, indicating that the 5′ end of BLV sequence was truncated (Fig. 3D and Table S4), whereas the other (IS2) contained complete virus sequence (Table S4). OCI of AS and PL-1 samples were relatively low, while that of PL-2 and PL-3 were high because of the presence of the major populations (Fig. 3C).Together, the BLV DNA-capture-seq successfully identified ISs, the BLV genome deletant and BLV full length consensus sequences in AS and PL cattle samples.

### SNP detection of BLV infected cattle

**Figure 4:**
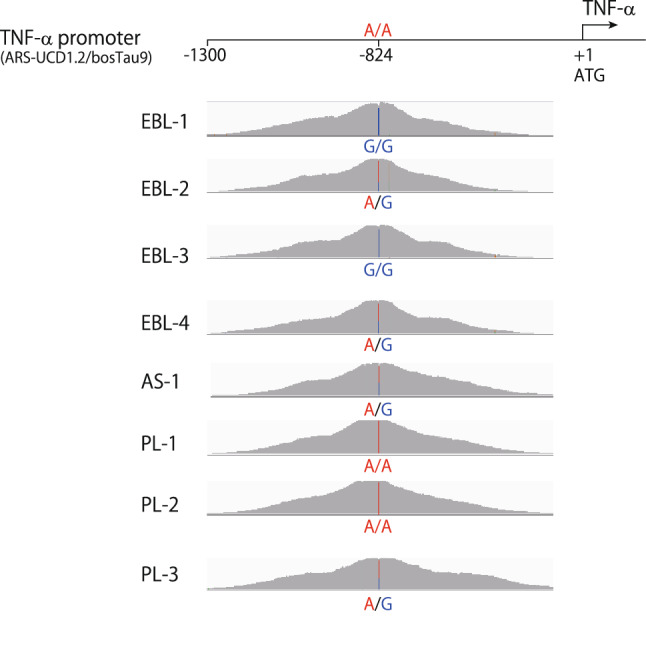
Amino acid polymorphisms of TNF-α promoter region. The sequence reads mapped to reference sequence (ARS-USD1.2/bosTau9) were visualized. Numbers denote the nucleotides count from the transcription initiation site. The nucleotides in the polymorphic sites are shown.

Previous studies have demonstrated that a SNP in the TNF-α promoter region at -824G/G is related to increased proviral load (PVL) because of the high promoter activity, which results in the progression of lymphoma^18,19^. To simultaneously analyze the SNP with ISs, clonal expansion and whole BLV proviral sequences, the complementary pair of probes targeted to the TNF-α promoter region were added to the probe pools. The probe sequences are shown on Table S2. The results of the enriched DNA sequences were successfully analyzed (Fig. 4). The sequence results revealed that -824G allele frequency was higher than that of TNF-α-824A allele in EBL cattle samples (75% for TNF-α-824G). In contrast, TNF-α-824G allele frequency was lower than that of TNF-α-824A allele in AS and PL cattle samples (25% for TNF-α-824G).

## Discussion

The target enrichment approach is a highly comprehensive, quantitative and sensitive method for NGS analysis of integrated retroviruses. We previously reported a significant increase of sensitivity in detecting HIV-1 and HTLV-1 proviral sequences by DNA-probe base enrichment^27–29^. Based on these previous studies, here we applied the DNA-capture-seq approach for the analysis of the biology of BLV infection. This method enables the simultaneous analysis of BLV whole proviral sequences, ISs, clonal abundance and a host SNP in one step.

Several previous studies have demonstrated BLV whole genome sequences using the amplicon sequencing method^9,12,28,30^. In this method, DNA fragments were produced by the standard PCR method with BLV-specific overlapping primers prior to Sanger sequencing or NGS. This method generates a pure amplicon of the viral template, which enables efficient sequencing of the proviral genome. A slight drawback exists in that the primer annealing regions set at the 5′ end and 3′ end of the proviral genome could not be applied for sequencing. Whole genome sequencing of BLV positive samples by NGS will solve this problem. However, due to the small size of the BLV proviral genome compared to the host genome, a high sequencing depth is needed to acquire substantial reads to determine the BLV proviral sequence. In this study, by using DNA capture probes specific to the BLV proviral genome, 10.2% of the total 0.97 million raw reads mapped on the target BLV reference genome. Compared to our previous study, in which 4.9% of the total 1.06 million raw reads mapped on the target virus genome of HIV-1 capture-seq, sequence method in this study proved to be highly efficient in BLV target enrichment^27^. There was a previous study that reported BLV whole genome sequencing or genotype 1 and 6 through another enrichment approach using the SureSelect system (Agilent)^31^, although the authors did not report the probe sequences, efficiency of enrichment, or coverage of the whole proviral genome. Other groups reported full length BLV sequences of genotype 1–4, 6, 9 and 10, by PCR amplification with Sanger sequencing or NGS. However, most of the reported sequences are genotype 1, and whole genome information of genotype 5, 7 and 8 have not yet been identified. The DNA-capture-seq approach in this study utilized probe pools containing individually synthesized probes (xGen Lockdown Probes, IDT) which enabled higher sequencing coverage throughout the whole BLV proviral genome and sensitive detection of low frequency variants. This sequence method currently covers BLV genotype 1 (FLK-BLV) and genotype 3 (newly sequenced in this study), which are the most common BLV genotypes in Japan, USA and Korea^6^. In our HTLV-1 DNA-capture-seq approach previously reported, less than 10% of mismatched nucleotides per probe did not significantly affect the enrichment step^27^. Among 58 representative BLV full length sequences, the maximum nucleotide difference between the reference sequence for probe (EF600696) was 458 bp/8720 bp (5.3%) (vs MF580992, genotype 6) (data not shown). The average nucleotide difference between each probe and BLV sequence appear to be less than 10%. Therefore, these probes are potentially feasible to enrich previously reported BLV sequences. While overall similarity of BLV genotypes is high, this method may be applicable to examine BLV genotypes other than genotype 1 and 3.

Importantly, in this study we present the evidence for the first time that 5′ defective BLV provirus was present in the PL cattle (Fig. 3D, PL-2). BLV is a member of the genus Delta retrovirus, which also includes HTLV-1. During HTLV-1 infection in vivo, 5′LTR-deleted defective HTLV-1 proviruses are often reported in ATL patients, AS carriers and HAM/TSP patients^32–34^. Regarding BLV, so far, there have been 81 complete BLV proviral genome sequences registered to public genome databases and defective provirus have been rarely reported^35^. The possible explanation for the absence of defective BLV provirus is that most of the BLV genome sequences were amplified by PCR with overlapping primers prior to Sanger sequencing. Indeed, in our data of consensus sequence of PL-2 sample, there were no obvious deletion detected in 5′ end of the proviral sequence because there are two other clones with full length proviruses within the same sample (Fig. 3C and Table S4). Also, the previous studies were limited to sequence analysis of the most abundant BLV genomes because of the insensitivity to detecting low-frequency variants by the PCR-based method. The ability of DNA-capture-seq to detect low-frequency variants of the BLV genome has been shown in the analysis of the FLK-BLV cell line (Table 1). A limitation of the method in this study for detecting BLV ISs includes an inability to detect deletion in the middle of the proviral genome if the sample contains multiple copies of BLV proviruses with complement sequences. Taken together, our high-resolution data of BLV DNA-capture-seq suggest that the defective BLV provirus genome should be taken into consideration for the pathogenesis of BLV.

Previously, BLV ISs were analyzed by inverse PCR and by ligation-mediated PCR^20,21,25^. The inverse PCR is a PCR assay for rapid amplification of the host genome sequences flanking a specific viral sequence by using inversely oriented specific primers. Inverse PCR involves restriction enzyme digestion and ligation for generating circular DNA before amplification of the host and virus chimeric region. Therefore, the data obtained by such assay may be limited due to the inefficiency of intramolecular ligation and amplification of circularized DNA. On the other hand, ISs analyzed by ligation-mediated PCR are highly specific, but require ligation, DNA purification and multiple PCR reactions which are quite time-consuming. In our DNA-capture-seq approach, the probes set at the end of the 5′ and 3′ end of LTR efficiently enrich chimeric reads of the BLV provirus and host genome, which results in the sensitive and rapid detection of BLV ISs. Through the analysis of the variation of the host sequences adjacent to the BLV genome, DNA-capture-seq also enables quantification of clonal expansion of BLV-infected cells. Previous studies demonstrated that BLV preferably integrated near CpG islands, or near cancer driver genes^21,25^. The clonality of EBL cattle was quite high in that one of the samples were monoclonal, and the other three samples were oligoclonal with two or three ISs (Fig. 3C and Table 2). Previous studies indicated that BLV-infected cells of most EBL cattle display monoclonal profiles^25,26^. However, in our results, EBL cattle with oligoclonal profiles exist in a higher proportion to cattle with monoclonal profiles. Further experimentation with larger sample sizes is required to estimate the proportion of EBL cattle with oligoclonal proviruses.

The mechanisms of lymphoma development after BLV infection are not fully elucidated. Viral products such as Tax and ASs are thought to play significant roles in oncogenic mechanisms. In addition, previous studies have demonstrated that polymorphisms in the host genomes involved in the immune response to the BLV affect the proviral load and risk of developing lymphoma^15–19^. Moreover, BLV integration into the host genome itself would accelerate the proliferation of infected cells^20,21^. The oncogenic processes of each factor have been extensively studied; however, the comprehensive understanding of these factors is limited. In our study, use of the complementary pair of probes set to the TNF-α promoter region allowed us to successfully enrich and detect host SNP simultaneously with ISs, clonal expansion and whole BLV proviral sequences. Previous study has shown that the frequency of the TNF-α-824 G allele is higher in cattle with EBL in association with low transcription activity of the promoter region of the bovine TNF-α gene expression. In our DNA-capture-seq data, TNF-α-824G allele frequency was also higher than that of TNF-α-824A allele in EBL cattle samples (75% for TNF-α-824G). In contrast, TNF-α-824G allele frequency was lower than that of TNF-α-824A allele in AS and PL cattle samples (25% for TNF-α-824G). Because of a small sample size, significant differences in allele frequency among pathogenetic stages could not be definitively elucidated. However, these data partially support the previous reports of the relationships between lymphoma development and polymorphisms in the TNF-α promoter region. The work present here is a pilot study, yet novel approach, based on a selection of a single SNP. The use of specific probes matching additional SNPs such as p53 tumor suppressor gene or tumor necrosis factor receptor type II gene (TNF-RII), previously reported to be associated with EBL pathogenesis, will make this approach more informative^36,37^.

In summary, we explored the DNA-capture-seq approach which enables comprehensive analysis of the BLV whole genome sequence, ISs, clonal expansion of infected cells as well as SNPs of the host genome. Analysis of four EBL cattle infected with BLV genotype 3 identified high clonality of infected cells and high allele frequency of TNF-α-824G. ISs of EBL-1 and EBL-4 were located next to genomic repeats while some ISs of EBL-2 and EBL-3 were located nearby transcribed regions. In contrast, analysis of AS and PL cattle infected with BLV genotype 1 showed polyclonal expansion of BLV infected cells, with an increase of major clone population partially associated with PVL. In addition, the defective provirus with higher resolution was detected in PL cattle. TNF-α-824G allele frequency was lower in AS and PL cattle group. These results demonstrated the effectiveness of this approach in analysis of EBL tumor tissue samples and BLV infected AS and PL blood samples. The efficient and sensitive identification of ISs would allow detection of low frequency clones, which could lead to a better understanding of polyclonal expansion of BLV-infected cells in early infection and during persistent infection together with the BLV proviral genome and host genome variability. The DNA-capture-seq approach described in this study has potential as an innovative tool for understanding the detailed mechanisms of BLV infection in the course of disease progression.

## Materials and methods

### DNA probes

One hundred and forty-five xGen Lockdown Probes (IDT) were custom-designed based on the BLV proviral sequence, accession number EF600696 (the list of probes can be found as Table S2). Probes were also custom-designed based on bovine tumor necrosis factor alpha (TNF-α) gene promoter sequence of *Bos Taurus* genome sequence (ARS-UCD1.2/bosTau9).

### Sample collection, PVL measurement AND genotype analysis

The persistently BLV-infected fetal lamb kidney cell line (FLK-BLV) established by Van Der Maaten and Miller^24^, was kindly provided by Dr. Yoko Aida, Institute of Physical and Chemical Research (RIKEN), Japan. Tumor samples were collected from cattle diagnosed as EBL by veterinary officers at the Meat Inspection Station of Kanagawa Prefectural Government. Heart is the most common organ that develop lymphoid neoplasm in EBL cattle and to acquire the comparative data, we selected heart for the analyses in this study. Genomic DNA was extracted from the samples using a commercially available kit (DNeasy Blood & tissue Kit: QIAGEN). The BLV PVL in tumor samples and blood samples were measured by quantitative-real time PCR using CoCoMo-qPCR kit (Riken Genesis), following the manufacturer’s instructions. BLV genotype was investigated by PCR–RFLP method. The detailed procedures were described in our previous paper^38^. Among 144 cattle samples, 140 samples were classified as genotype 1 and 4 samples were classified as genotype 3. The numbers of leukocytes and lymphocytes in EDTA-treated whole blood were quantified using a hematology analyzer (Erma Inc). BLV-infected cattle were classified as PL if they met the criteria of Bendixen’s key^39^. BLV-infected cattle that did not fulfill Bendixen’s key were classified as AS.

### Library synthesis and targeted enrichment

Construction of DNA libraries and enrichment of targeted fragments were performed as previously described^29,33^. Briefly, DNAs were fragmented by Picoruptor (Diagnode Diagnostics), and DNA libraries for NGS were prepared with the NEBNext Ultra II DNA Library Prep Kit and NEBNext Multiplex Oligos for Illumina (New England BioLabs). Multiplexed libraries were subjected to cluster generation using a NextSeq 500/550 Mid Output Kit v2.5 (150 cycles) in NextSeq desktop sequencing systems (Illumina). In order to reach the recommended amount of 1000 ng, up to 5 samples containing different index sequences were combined. The xGen Lockdown Reagents kit (IDT) was used for hybridization with the virus-specific probes to perform the enrichment step, following instructions provided by the manufacturer. The DNA library was mixed with 5 μg of human Cot-1 DNA (Invitrogen) and xGen Universal Blocking Oligos (IDT), and then dried using an evaporator (Eyela). The dried DNA was dissolved in the hybridization buffer, followed by an incubation step at 95 °C for 10 min. The probes were added and hybridized at 65 °C for 4 h, then streptavidin-coated magnetic beads (Dynabeads M-270 Streptavidin, Thermo Fisher Scientific) were further added and incubated at 65 °C for 45 min. The captured DNA was washed and amplified by PCR, and further purified using Agencourt AMPure XP beads (Beckman Coulter). Prior to the sequencing step, DNA libraries enriched for proviral sequences were quantified by Tapestation instrument (Agilent Technologies), and qPCR (GenNext NGS library quantification kit, Toyobo) using Illumina’s adaptor binding sites P5 and P7.

### High throughput sequencing data analysis

The obtained data from Illumina NextSeq include FASTQ files of Read1, Read2 and Index Read. Cutadapt and PRINSEQ ver0.20.4 were used for a data-cleaning step, and obtained sequences were aligned to *Bos Taurus* reference genome sequence (ARS-UCD1.2/bosTau9) with BLV (GenBank: EF600696) as a separate chromosome or integrated provirus using the BWA-MEM algorithm^40^. The sequence reads with multiple alignments and duplications were further processed and cleaned up with Samtools^40^ and Picard (http://broadinstitute.github.io/picard/). The aligned reads were visualized by Integrative Genomics Viewer (IGV)^41^.

### IS and proviral structure analysis with DNA-seq data

The cleaned FASTQ files were aligned to the bovine reference genome (chr 1–29 and X) and BLV proviral genome. For the BLV proviral genome, the reference sequence was divided into 2 parts: the viral LTR sequence (BLV_LTR) and the whole viral sequence excluding the LTRs (BLV_noLTR). Since BLV target probes capture not only BLV sequence reads but also chimeric reads containing both the BLV and host genome, the virus-host chimeric reads were extracted for determining the locations of BLV IS in the host genome using the method we previously described^27,29,33^. The clonal abundance for each infected clone was quantified by the number of virus-host reads. The number of final virus-host reads in a certain genomic region reflects the initial cell number of the clone. That would enable us to estimate the initial copy number of each clone by counting the number of virus-host reads after removal of PCR replicates as previously reported^33^.

### PCR amplification of viral-host junctions

To confirm detected ISs, nucleotide sequences were amplified by PCR using primers set to the host genome and BLV genome, and Ex-taq (Takara Bio Inc.). Primers were listed in Table S3 The reaction mixture contained approximately 400 ng of template DNA, 5 μl of 10 × Ex Taq Buffer, 4 μl of 2.5 mM dNTP mix, 0.25 μl of Ex Taq polymerase, and 1 μl of each primer (10 μM) in a total of 50 μl of aliquots. Conditions for PCR amplification were as follows: 30 cycles of denaturation at 98 °C for 10 s, annealing at 58 °C for 30 s, and extension at 72 °C for 20 s. Amplified products were visualized on 1% agarose gels (Takara Bio Inc.)

### Phylogenetic analysis

Phylogenetic analysis of the assembled BLV genomes were conducted using MEGA version 7^42^. The evolutionary history was inferred by use of the Maximum Likelihood method based on the Kimura 2-parameter model^43^. Evolutionary analyses were conducted in MEGA7 and MEGA X^42,44^.

### Estimates of evolutionary divergence over sequence pairs between groups

The number of base differences per site from averaging over sequence pairs between genotypes was calculated. Codon positions included were 1st + 2nd + 3rd + Noncoding. All ambiguous positions were removed for each sequence pair (pairwise deletion option). Evolutionary analyses were conducted in MEGA X^44^.

### Detection of positive selection sites

To analyze the presence of positive selection selective pressure in BLV genotype3, phylogenetic trees were generated by BEAST v1.8.3. We used the General Time Reversible (GTR) with gamma-distributed rate variation across sites and a proportion of invariant sites. Positive selective pressure of six ORFs (*G4*, *gag*, *pro*, *pol*, *env* and *tax*) of BLV genotype 3 were analyzed by CODEML program in the PAML (Phylogenetic Analysis by Maximum. Likelihood) package v4.8 with the branch site model of codon-based likelihood methods^45^. In these analyses, maximum likelihood estimates of the positive selection pressure were based on the ratio of non-synonymous to synonymous substitution (dN/dS). The dN/dS and likelihood scores were calculated for two branch site models (MA and MA1): MA allows positive selection on one or more lineages (called the foreground branch), while MA1 is a null model (which does not allow positive selection). The LRT was used to compare the two nested models which, based on twice the log likelihood difference between the two models (2∆lnL), follows a χ2 distribution with one degree of freedom. Sites under positive selection pressure on the foreground branch were identified by using the BEB method, if the LRTs are statistically significant.

### Ethical approval

This study was approved by the animal research committee at Tokyo University of Agriculture, and we confirm that all experiments were performed in accordance with the committee’s guidelines and regulations.

## Supplementary Information

Supplementary Information
