# Supplementary material for: A target enrichment high throughput sequencing system for characterization of BLV whole genome sequence, integration sites, clonality and host SNP
Source: Sci Rep. 2021 Feb 25;11:4521. doi: 10.1038/s41598-021-83909-3 (PMC7907107; doi:10.1038/s41598-021-83909-3)
Supplement: Supplementary file 1 — Supplementary Information [file 41598_2021_83909_MOESM1_ESM.pdf]

A target enrichment high through-put sequencing system for characterization of BLV whole genome sequence, integration sites, clonality and host SNP

Nagaki Ohnuki, Tomoko Kobayashi, Misaki Matsuo, Kohei Nishikaku, Kazuya Kusama, Yasushi Torii, Yasuko Inagaki, Masatoshi Hori, Kazuhiko Imakawa and Yorifumi Satou

Supplementary Information:

Supplementary Figures
Fig. S1: Sequence characterization of BLV strains analyzed in this study.
Fig. S2: Maximum-likelihood phylogenetic tree analysis of BLV AS1, G4 and tax.
Fig. S3: Full length gel image of Figure 2B
Fig. S4: Full length gel image of Figure 3B

Supplementary Tables
Table S1. Summary of characteristics of cattle analyzed in this study.
Table S2: List of probes used for the capture of BLV provirus and host genome
Table S3. List of primers used for the PCR of BLV provirus and host genome amplification.
Table S4. The list of virus-host reads detected in blood samples of AS and PL cattle.

A

		Sequence Identity									
		EF600696	FLK-BLV	AS1	PL1	PL2	PL3	EBL1	EBL2	EBL3	EBL4
Sequence Difference Count	EF600696		0.999	0.993	0.993	0.993	0.993	0.972	0.971	0.97	0.972
	FLK-BLV	3		0.992	0.992	0.993	0.993	0.971	0.971	0.97	0.972
	AS1	61	64		1	0.994	0.996	0.971	0.97	0.97	0.971
	PL1	61	64	0		0.994	0.996	0.971	0.97	0.97	0.971
	PL2	54	57	45	45		0.994	0.972	0.971	0.971	0.972
	PL3	58	61	27	27	44		0.971	0.97	0.97	0.971
	EBL1	244	247	248	248	242	250		0.998	0.998	0.991
	EBL2	249	248	253	253	247	255	11		0.998	0.991
	EBL3	253	254	257	257	251	259	13	10		0.99
	EBL4	243	244	245	245	237	247	75	76	80	

B

	GAG									PRO		POL												
a.a.position	61	63	87	92	108	318	323	365	391	91	134	40	97	210	423	437	453	481	517	570	577	602	774	830
EF600696	G	T	D	G	V	V	V	A	S	V	V	A	P	V	D	T	Q	V	N	S	V	R	E	L
FLK-BLV	·	·	·	·	·	·	·	·	·	·	·	·	·	·	·	·	·	·	·	·	·	·	·	·
AS1	·	·	E	·	·	M	I	·	·	·	·	·	S	·	·	·	·	·	S	·	I	K	·	·
PL2	·	·	E	·	·	M	I	·	·	·	·	·	S	·	·	·	·	·	S	·	I	K	·	·
PL3	·	·	E	·	·	M	I	·	·	·	·	·	S	·	·	·	·	·	S	·	I	K	·	·
PL4	·	·	E	·	I	M	I	·	·	·	·	·	S	·	·	K	·	·	S	·	I	·	·	·
EBL1	S	A	·	D	·	I	I	T	·	I	A	T	S	·	E	·	·	L	S	A	I	K	·	P
EBL2	S	A	·	D	·	I	I	T	·	I	A	T	S	·	E	·	·	L	S	A	I	K	·	P
EBL3	S	A	·	D	·	I	I	T	·	I	A	T	S	·	E	·	·	L	S	A	I	K	·	P
EBL4	S	A	·	·	·	I	I	T	P	I	A	T	S	I	E	·	R	L	S	A	I	K	D	P

	ENV																				R3				
	4	28	29	47	58	79	80	82	107	133	141	254	337	466	469	477	479	480	500	504	4	22	31	34	44
EF600696	K	C	R	T	S	T	L	S	T	A	N	S	H	K	R	I	L	T	H	V	K	V	R	S	F
FLK-BLV	·	·	·	·	·	·	·	·	·	·	·	·	·	·	·	·	·	·	·	·	·	·	·	·	·
AS1	E	S	·	·	·	A	·	·	·	·	·	·	·	·	·	·	·	·	·	·	E	·	·	·	·
PL2	E	S	·	·	·	A	·	·	·	·	·	·	·	·	·	·	·	·	·	·	E	·	·	·	·
PL3	E	·	·	·	P	·	·	·	·	·	·	·	·	·	·	·	·	·	·	·	E	·	·	·	·
PL4	E	S	·	·	·	·	·	·	·	·	·	·	·	·	·	T	·	·	·	·	E	·	·	·	·
EBL1	E	·	Q	I	·	·	M	F	·	T	D	L	·	R	Q	·	F	A	R	T	E	I	S	·	L
EBL2	E	·	Q	I	·	·	M	F	·	T	D	L	·	R	Q	·	F	A	R	T	E	I	S	·	L
EBL3	E	·	Q	I	·	·	M	F	·	T	D	L	R	R	Q	·	F	A	R	T	E	I	S	·	L
EBL4	E	·	Q	·	·	·	·	F	A	T	D	L	·	·	Q	·	F	A	R	T	E	·	·	G	L

	G4								Rex														
	25	35	37	40	41	52	60	103	4	35	38	52	58	59	67	72	75	80	115	120	141	146	157
EF600696	Q	F	T	I	K	H	R	F	K	F	M	M	K	G	R	T	P	A	S	H	H	L	*
FLK-BLV	·	·	·	·	·	·	·	·	·	·	·	·	·	·	·	·	·	·	·	·	·	·	S
AS1	·	·	I	·	·	L	·	·	E	·	·	·	·	·	·	·	L	·	·	·	·	·	·
PL2	·	·	I	·	·	L	·	·	E	·	·	·	·	·	·	·	L	·	·	·	·	·	·
PL3	·	·	·	·	·	·	·	·	E	·	·	·	·	·	·	·	·	·	·	Y	Y	·	·
PL4	·	·	·	·	·	L	·	·	E	·	·	·	E	·	·	·	·	·	·	·	·	·	·
EBL1	·	S	·	R	E	·	Q	·	E	·	·	T	·	R	K	P	·	S	N	R	R	S	·
EBL2	·	S	·	R	E	·	Q	·	E	·	·	T	·	R	K	P	·	S	N	R	R	S	·
EBL3	·	S	·	R	E	·	Q	·	E	·	·	T	·	R	K	P	·	S	N	R	R	S	·
EBL4	R	S	·	R	E	·	Q	L	E	S	V	·	·	R	K	P	·	S	·	R	R	S	·

	TAX																	AS1						
	22	42	43	56	64	104	105	141	148	164	185	194	222	233	257	289	305	3	16	21	25	51	66	81
EF600696	N	E	R	N	C	S	L	L	R	S	A	T	N	L	C	I	L	P	E	D	A	S	L	E
FLK-BLV	·	·	·	·	·	·	·	F	·	·	·	·	·	·	·	·	·	·	·	·	·	·	·	·
AS1	·	·	·	·	·	·	·	·	G	·	·	M	·	·	·	·	·	·	·	E	·	·	·	·
PL2	·	·	·	·	·	·	·	·	G	·	·	M	·	·	·	·	·	·	·	E	·	·	·	·
PL3	·	·	·	·	·	L	·	·	G	·	·	·	·	·	·	·	·	·	·	·	·	·	·	·
PL4	·	G	·	·	·	·	·	·	G	·	·	·	·	·	·	·	·	L	·	E	·	·	·	·
EBL1	·	·	K	T	F	·	F	·	G	P	T	·	S	P	·	L	I	·	A	·	G	A	F	G
EBL2	·	·	K	T	F	·	F	·	G	P	T	·	S	P	·	L	I	·	A	·	G	A	F	G
EBL3	·	·	K	T	F	·	F	·	G	P	T	·	S	P	·	L	I	·	A	·	G	A	F	G
EBL4	S	·	K	T	F	·	·	·	G	P	T	·	·	·	Y	L	I	·	A	·	G	·	F	G

Fig. S1: Sequence characterization of BLV strains analyzed in this study.
(A) Pairwise sequence identity and different nucleotide. (B) Alignment of amino acid sequences of BLV proteins. Alignment of amino acid residues corresponding to virus proteins from a total 9 BLV consensus sequences analyzed in this study, together with that of FLK-BLV strain pBLV913.

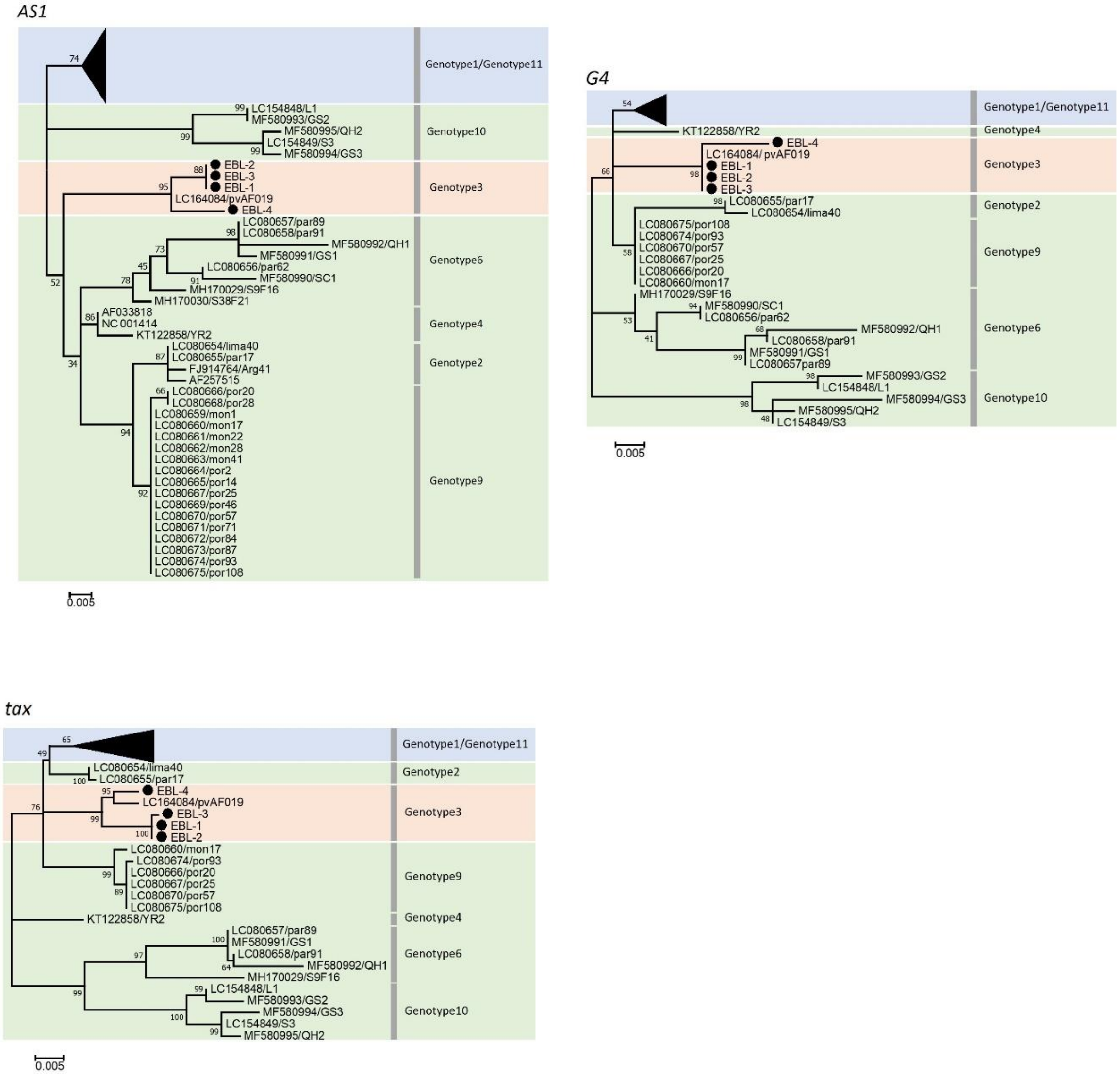

Fig. S2: Maximum-likelihood phylogenetic tree analysis of BLV AS1, G4 and tax. Sequences were generated with four newly obtained sequences from EBL tumor samples (sequences indicated by ●) together with sequences from the GenBank database (117 sequences of *AS1*, 54 sequences of *G4*: and 53 sequences of *tax*). The phylogenetic tree was generated and visualized using MEGA 7 with 1,000 bootstrap replicates. Collapsed branches represent genotype 1 and 11. The bar at the bottom of the figure denotes the estimated number of amino acid substitutions per site, indicating genetic variation for the length of the scale.

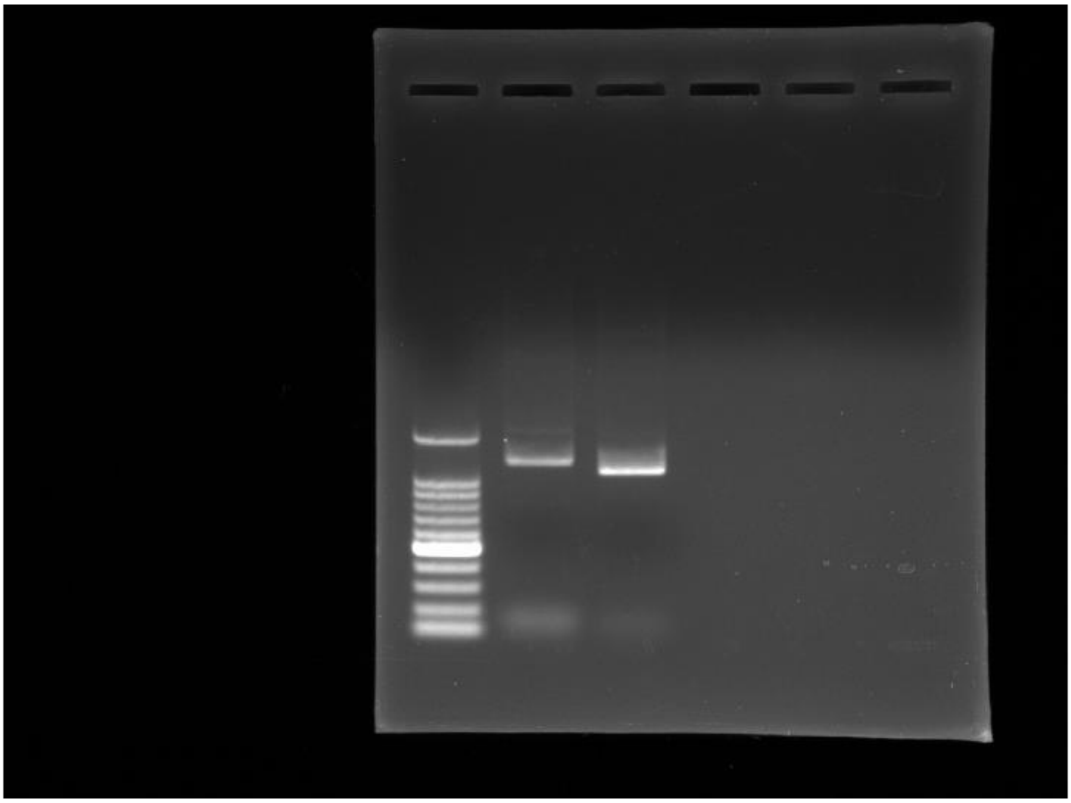

Fig. S3: full length gel image of Figure 2B

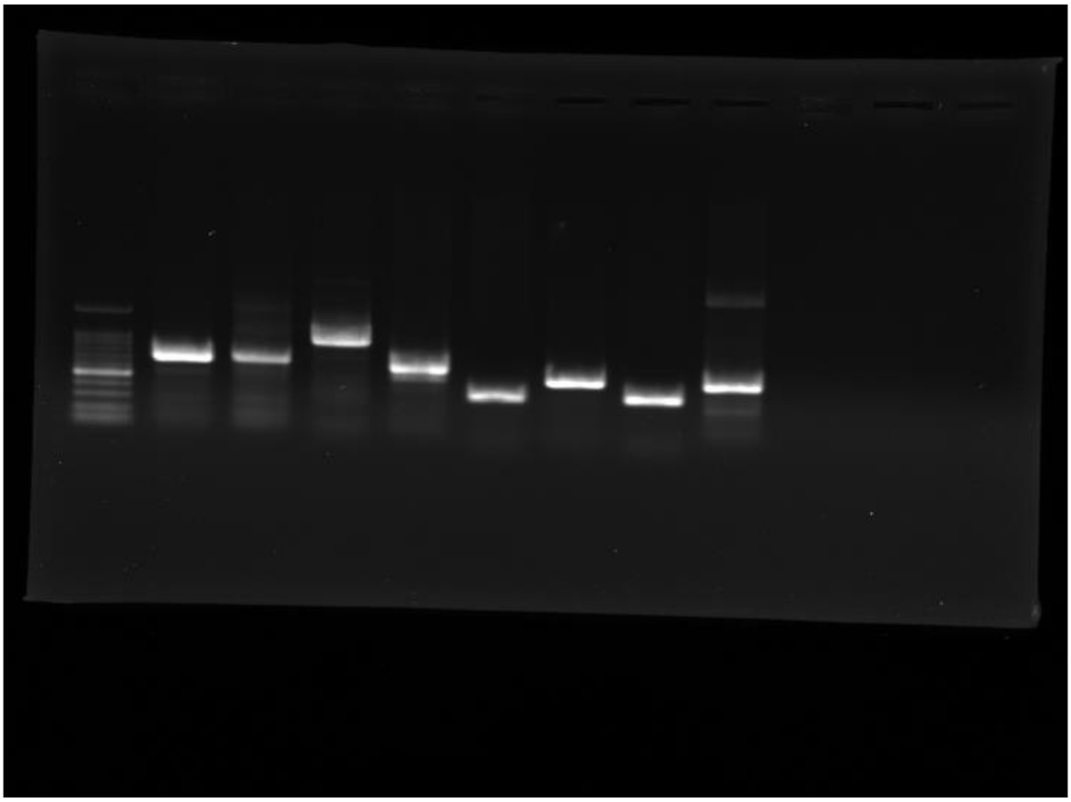

Fig. S4: full length gel image of Figure 3B

Table S1. Summary of characteristics of cattle analyzed in this study.

Cattle No.	Breed	Age, y	PVL(copies/10^5cells)	PBMCs(x10^3 /μl)	Place of birth	tissues with lymphoid tumors
EBL1	Holstein	3	883,333	NA	Kanagawa	heart, kidneys, lung, rumen, uterus, intestine, lymph nodes
EBL2	Holstein	7	364,696	NA	Hokkaido	heart, lung, rumen, uterus, lymph nodes
EBL3	Holstein	4	294,185	NA	Kanagawa	heart, kidneys, lung, rumen, uterus, intestine, lymph nodes
EBL4	Holstein	5	106,783	NA	Shizuoka	heart, kidneys, rumen, uterus, intestine, lymph nodes
AS	Holstein	4	37,494	6.5	Hokkaido	-
PL-1	Holstein	7	51,576	12.8	Hokkaido	-
PL-2	Holstein	6	72,947	10.6	Kanagawa	-
PL-3	Holstein	11	89,911	13.4	Kanagawa	-

underlined tumor samples were analyzed in this study

NA; not available

PVL; proviral load

- ; not applicable

Table S2. List of probes used for the capture of BLV provirus and host genome

target (reference)	Probe sequence	Start	End
BLV proviral genome (EF600696)			
1	TGTATGAAAGATCATGCCGACCTAGGCGCCGCCACCGCCCCGTAAACCAGACAGAGACGTCAGCTGCCAGAAAAGCTGGTGACGGCAGCTGGTGGCTAGAATCCCCGTACCTCCCCAACT	0	120
2	CAGCTGCCAGAAAAGCTGGTGACGGCAGCTGGTGGCTAGAATCCCCGTACCTCCCCAACTTCCCCTTTCCCGAAAAATCCACACCCTGAGCTGCTGACCTCACCTGCTGATAAATTAATA	60	180
3	TCCCCTTTCCCGAAAAATCCACACCCTGAGCTGCTGACCTCACCTGCTGATAAATTAATAAAATGCCGGCCCTGTCGAGTTAGCGGCACCAGAAGCGTTCTTCTCCTGAGACCCTCGTGC	120	240
4	AAATGCCGGCCCTGTCGAGTTAGCGGCACCAGAAGCGTTCTTCTCCTGAGACCCTCGTGCTCAGCTCTCGGTCCTGAGCTCTCTTGCTCCCGAGACCTTCTGGTCGGCTATCCGGCAGCG	180	300
5	TCAGCTCTCGGTCCTGAGCTCTCTTGCTCCCGAGACCTTCTGGTCGGCTATCCGGCAGCGGTCAGGTAAGGCAAACCACGGTTTGGAGGGTGGTTCTCGGCTGAGACCACCGCGAGCTCT	240	360
6	GTCAGGTAAGGCAAACCACGGTTTGGAGGGTGGTTCTCGGCTGAGACCACCGCGAGCTCTATCTCCGGTCCTCTGACCGTCTCCACGTGGACTCTCTCCTTTGCCTCCTGACCCCGCGCT	300	420
7	ATCTCCGGTCCTCTGACCGTCTCCACGTGGACTCTCTCCTTTGCCTCCTGACCCCGCGCTCCAAGGGCGTCTGGCTTGCACCCGCGTTTGTTTCCTGTCTTACTTTCTGTTTCTCGCGGC	360	480
8	CCAAGGGCGTCTGGCTTGCACCCGCGTTTGTTTCCTGTCTTACTTTCTGTTTCTCGCGGCCCGCGCTCTCTCCTTCGGCGCCCTCTAGCGGCCAGGAGAGACCGGCAAACAATTGGGGGC	420	540
9	CCGCGCTCTCTCCTTCGGCGCCCTCTAGCGGCCAGGAGAGACCGGCAAACAATTGGGGGCTCGTCCGGGATTGATCACCCCGGAACCCTAATAACTCTCTGGACCCACCCCCTCGGCGGC	480	600
10	TCGTCCGGGATTGATCACCCCGGAACCCTAATAACTCTCTGGACCCACCCCCTCGGCGGCATTTTGGGTCTCTCCTTCAAATTATATCATGGGAAATTCCCCCTCCTATAACCCCCCCGC	540	660
11	ATTTTGGGTCTCTCCTTCAAATTATATCATGGGAAATTCCCCCTCCTATAACCCCCCCGCTGGTATCTCCCCCTCAGACTGGCTCAACCTTCTGCAAAGCGCGCAAAGGCTCAATCCGCG	600	720
12	TGGTATCTCCCCCTCAGACTGGCTCAACCTTCTGCAAAGCGCGCAAAGGCTCAATCCGCGACCCTCTCCCAGCGATTTTACCGATTTAAAGAATTACATCCATTGGTTTCATAAGACCCA	660	780
13	ACCCTCTCCCAGCGATTTTACCGATTTAAAGAATTACATCCATTGGTTTCATAAGACCCAGAAAAAACCATGGACTTTCACTTCTGGTGGCCCCACCTCATGTCCACCCGGGAGATTCGG	720	840
14	GAAAAAACCATGGACTTTCACTTCTGGTGGCCCCACCTCATGTCCACCCGGGAGATTCGGCCGGGTTCCCCTTGTCTTGGCCACCCTAAACGAAGTGCTCTCAAACGATGGGGGCGCCCC	780	900
15	CCGGGTTCCCCTTGTCTTGGCCACCCTAAACGAAGTGCTCTCAAACGATGGGGGCGCCCCGGGTGCATCGGCCCCAGAAGAACAACCCCCCCCTTATGACCCCCCCGCCGTTTTGCCAAT	840	960
16	GGGTGCATCGGCCCCAGAAGAACAACCCCCCCCTTATGACCCCCCCGCCGTTTTGCCAATCATATCTGAAGGGAATCGCAACCGCCATCGTGCTTGGGCACTCCGAGAATTACAAGATAT	900	1020
17	CATATCTGAAGGGAATCGCAACCGCCATCGTGCTTGGGCACTCCGAGAATTACAAGATATCAAAAAGGAAATTGAAAATAAGGCACCGGGTTCGCAAGTATGGATACAAACACTACGACT	960	1080
18	CAAAAAGGAAATTGAAAATAAGGCACCGGGTTCGCAAGTATGGATACAAACACTACGACTTGCAATCCTGCAGGCCGACCCTACTCCGGCTGACCTAGAACAACTTTGCCAATATATTGC	1020	1140
19	TGCAATCCTGCAGGCCGACCCTACTCCGGCTGACCTAGAACAACTTTGCCAATATATTGCTTCCCCGGTCGACCAAACGGCCCATATGACCAGCCTAACGGCAGCAATAGCCGCCGCTGA	1080	1200
20	TTCCCCGGTCGACCAAACGGCCCATATGACCAGCCTAACGGCAGCAATAGCCGCCGCTGAAGCGGCCAACACCCTCCAGGGTTTTAACCCCCAAAACGGGACCCTAACCCAACAATCAGC	1140	1260
21	AGCGGCCAACACCCTCCAGGGTTTTAACCCCCAAAACGGGACCCTAACCCAACAATCAGCTCAGCCCAACGCCGGGGATCTTAGAAGTCAATATCAAAACCTCTGGCTTCAGGCCTGGAA	1200	1320
22	TCAGCCCAACGCCGGGGATCTTAGAAGTCAATATCAAAACCTCTGGCTTCAGGCCTGGAAAAATCTCCCTACTCGTCCTTCAGTACAACCTTGGTCCACCATCGTCCAAGGCCCCGCCGA	1260	1380
23	AAATCTCCCTACTCGTCCTTCAGTACAACCTTGGTCCACCATCGTCCAAGGCCCCGCCGAAAGCTATGTAGAGTTTGTCAACCGGTTACAAATTTCATTAGCTGACAACCTTCCCGACGG	1320	1440
24	AAGCTATGTAGAGTTTGTCAACCGGTTACAAATTTCATTAGCTGACAACCTTCCCGACGGAGTCCCTAAGGAACCCATTATTGACTCCCTTAGTTATGCAAATGCTAACAAAGAGTGCCA	1380	1500
25	AGTCCCTAAGGAACCCATTATTGACTCCCTTAGTTATGCAAATGCTAACAAAGAGTGCCAGCAAATTTTGCAGGGGCGAGGCCTAGTGGCCGCCCCGGTGGGGCAAAAACTGCAGGCTTG	1440	1560
26	GCAAATTTTGCAGGGGCGAGGCCTAGTGGCCGCCCCGGTGGGGCAAAAACTGCAGGCTTGCGCACATTGGGCCCCCAAGGTGAAACAGCCTGCAGTTCTCGTCCACACCCCAGGGCCCAA	1500	1620
27	CGCACATTGGGCCCCCAAGGTGAAACAGCCTGCAGTTCTCGTCCACACCCCAGGGCCCAAGATGCCCGGGCCTCGGCAACCGGCCCCCAAAAGGCCTCCCCCAGGACCATGCTATCGATG	1560	1680
28	GATGCCCGGGCCTCGGCAACCGGCCCCCAAAAGGCCTCCCCCAGGACCATGCTATCGATGCCTCAAAGAAGGCCATTGGGCCCGGGATTGTCCTACCAAGGCCACCGGCCCCCCTCCGGG	1620	1740
29	CCTCAAAGAAGGCCATTGGGCCCGGGATTGTCCTACCAAGGCCACCGGCCCCCCTCCGGGACCTTGCCCCATATGTAAAGATCCTTCCCATTGGAAACGAGACTGTCCAACCCTCAAATC	1680	1800
30	ACCTTGCCCCATATGTAAAGATCCTTCCCATTGGAAACGAGACTGTCCAACCCTCAAATCAAAAAACTAATAGAGGGGGGACTTAGCGCCCCCCAAACCATAACACCTATAACAGATTCT	1740	1860
31	AAAAAACTAATAGAGGGGGGACTTAGCGCCCCCCAAACCATAACACCTATAACAGATTCTCTTAGTGAGGCCGAATTAGAATGCTTACTTTCTATTCCTCTGGCTCGCAGCCGTCCCTCC	1800	1920
32	CTTAGTGAGGCCGAATTAGAATGCTTACTTTCTATTCCTCTGGCTCGCAGCCGTCCCTCCGTGGCTGTATACCTGTCTGGCCCCTGGCTGCAGCCCTCTCAGAATCAAGCCCTCATGCTT	1860	1980
33	GTGGCTGTATACCTGTCTGGCCCCTGGCTGCAGCCCTCTCAGAATCAAGCCCTCATGCTTGTGGACACCGGGGCTGAAAATACGGTTCTCCCACAAAATTGGCTGGTTCGAGATTACCCA	1920	2040
34	GTGGACACCGGGGCTGAAAATACGGTTCTCCCACAAAATTGGCTGGTTCGAGATTACCCACGGATCCCCGCCGCAGTGCTCGGAGCAGGGGGAGTCTCCCGGAACAGATACAATTGGCTA	1980	2100
35	CGGATCCCCGCCGCAGTGCTCGGAGCAGGGGGAGTCTCCCGGAACAGATACAATTGGCTACAAGGCCCTCTGACCCTGGCTCTAAAACCAGAGGGTCCCTTTATCACCATCCCAAAAATT	2040	2160
36	CAAGGCCCTCTGACCCTGGCTCTAAAACCAGAGGGTCCCTTTATCACCATCCCAAAAATTTTAGTTGACACTTTCGACAAATGGCAAATTTTAGGACGGGACGTCCTCTCCCGCCTACAG	2100	2220
37	TTAGTTGACACTTTCGACAAATGGCAAATTTTAGGACGGGACGTCCTCTCCCGCCTACAGGCCTCTATCTCCATACCTGAGGAAGTACGCCCCCCTGTGGTAGGCGTCTTGGATGCCCCC	2160	2280
38	GCCTCTATCTCCATACCTGAGGAAGTACGCCCCCCTGTGGTAGGCGTCTTGGATGCCCCCCCGAGCCACATTGGATTAGAACATCTGCCCCCCCCACCTGAGGTGCCTCAATTCCCTTTA	2220	2340
39	CCGAGCCACATTGGATTAGAACATCTGCCCCCCCCACCTGAGGTGCCTCAATTCCCTTTAAACTAGAACGCCTCCAGGCCCTTCAAGACCTGGTCCATCGCTCTCTGGAGGCAGGTTATA	2280	2400

40	AACTAGAACGCCTCCAGGCCCTTCAAGACCTGGTCCATCGCTCTCTGGAGGCAGGTTATATCTCCCCCTGGGACGGGCCAGGCAATAATCCAGTCTTCCCGGTACGGAAACCAAATGGCG	2340	2460
41	TCTCCCCCTGGGACGGGCCAGGCAATAATCCAGTCTTCCCGGTACGGAAACCAAATGGCGCCTGGAGGTTTGTGCATGACCTACGAGCTACAAATGCTCTTACAAAGCCCATTCCGGCAC	2400	2520
42	CCTGGAGGTTTGTGCATGACCTACGAGCTACAAATGCTCTTACAAAGCCCATTCCGGCACTCTCCCCCGGACCGCCAGACCTTACCGCTATCCCTACACACCTTCCACATATCATTTGCC	2460	2580
43	TCTCCCCCGGACCGCCAGACCTTACCGCTATCCCTACACACCTTCCACATATCATTTGCCTGGATCTCAAAGATGCCTTCTTCCAGATTCCAGTCGAAGACCGCTTCCGCCCCTACTTTG	2520	2640
44	TGGATCTCAAAGATGCCTTCTTCCAGATTCCAGTCGAAGACCGCTTCCGCCCCTACTTTGCTTTTACCCTCCCTACCCCCGGGGGACTCCAACCTCATAGACGCTTTGCCTGGCGGGTCC	2580	2700
45	CTTTTACCCTCCCTACCCCCGGGGGACTCCAACCTCATAGACGCTTTGCCTGGCGGGTCCTACCTCAAGGCTTCATTAACAGCCCAGCTCTTTTCGAACGAGCACTACAGGAACCTCTTC	2640	2760
46	TACCTCAAGGCTTCATTAACAGCCCAGCTCTTTTCGAACGAGCACTACAGGAACCTCTTCGTCAAGTATCAGCCGCCTTTTCCCAGTCTCTTCTGGTGTCCTATATGGACGATATCCTTA	2700	2820
47	GTCAAGTATCAGCCGCCTTTTCCCAGTCTCTTCTGGTGTCCTATATGGACGATATCCTTATCGCTTCGCCTACAGAAGAACAACGGTCACAATGTTATCAAGCCCTGGCTGCCCGCCTCC	2760	2880
48	TCGCTTCGCCTACAGAAGAACAACGGTCACAATGTTATCAAGCCCTGGCTGCCCGCCTCCGGGACCTAGGGTTTCAGGTGGCATCCGAAAAGACTCGCCAGACGCCTTCGCCCGTCCCCT	2820	2940
49	GGGACCTAGGGTTTCAGGTGGCATCCGAAAAGACTCGCCAGACGCCTTCGCCCGTCCCCTTCTTGGGACAAATGGTCCATGAGCAGATTGTCACCTACCAGTCCCTACCTACCTTGCAGA	2880	3000
50	TCTTGGGACAAATGGTCCATGAGCAGATTGTCACCTACCAGTCCCTACCTACCTTGCAGATCTCATCCCCAATTTCTCTTCACCAATTACAGGCGGTCTTAGGAGACCTCCAATGGGTCT	2940	3060
51	TCTCATCCCCAATTTCTCTTCACCAATTACAGGCGGTCTTAGGAGACCTCCAATGGGTCTCTAGGGGCACACCCACTACCCGCCGGCCCCTGCAACTTCTCTACTCTTCCCTTAAAGGCA	3000	3120
52	CTAGGGGCACACCCACTACCCGCCGGCCCCTGCAACTTCTCTACTCTTCCCTTAAAGGCATCGATGACCCTAGGGCCATCATCCAGCTTTCCCCGGAACAGCTGCAAGGCATTGCAGAGC	3060	3180
53	TCGATGACCCTAGGGCCATCATCCAGCTTTCCCCGGAACAGCTGCAAGGCATTGCAGAGCTTCGACAAGCCCTGTCCCATAACGCAAGATCTAGATATAACGAGCAAGAACCCCTGCTAG	3120	3240
54	TTCGACAAGCCCTGTCCCATAACGCAAGATCTAGATATAACGAGCAAGAACCCCTGCTAGCCTACGTACATCTAACCCGGGCGGGGTCCACCCTGGTACTCTTCCAAAAGGGCGCTCAAT	3180	3300
55	CCTACGTACATCTAACCCGGGCGGGGTCCACCCTGGTACTCTTCCAAAAGGGCGCTCAATTTCCCCTGGCCTACTTTCAGACCCCCTTGACTGACAACCAAGCCTCACCTTGGGGCCTCC	3240	3360
56	TTCCCCTGGCCTACTTTCAGACCCCCTTGACTGACAACCAAGCCTCACCTTGGGGCCTCCTTCTCCTGCTGGGATGCCAATACCTGCAGACTCAGGCCTTAAGCTCGTATGCCAAGCCCA	3300	3420
57	TTCTCCTGCTGGGATGCCAATACCTGCAGACTCAGGCCTTAAGCTCGTATGCCAAGCCCATACTTAAATATTATCATAATCTTCCTAAAACCTCTCTAGACAATTGGATTCAATCATCTG	3360	3480
58	TACTTAAATATTATCATAATCTTCCTAAAACCTCTCTAGACAATTGGATTCAATCATCTGAGGACCCTCGAGTCCAGGAGTTGCTGCAATTGTGGCCCCAGATTTCCTCTCAGGGAATAC	3420	3540
59	AGGACCCTCGAGTCCAGGAGTTGCTGCAATTGTGGCCCCAGATTTCCTCTCAGGGAATACAGCCCCCGGGCCCTTGGAAGACCTTAATCACCAGGGCAGAGGTTTTTTTGACGCCCCAGT	3480	3600
60	AGCCCCCGGGCCCTTGGAAGACCTTAATCACCAGGGCAGAGGTTTTTTTGACGCCCCAGTTCTCCCCTGATCCGATTCCTGCGGCCCTTTGCCTCTTTAGTGACGGGGCTACAGGACGAG	3540	3660
61	TCTCCCCTGATCCGATTCCTGCGGCCCTTTGCCTCTTTAGTGACGGGGCTACAGGACGAGGAGCATATTGCTTGTGGAAAGACCACCTTTTAGACTTTCAGGCCGTTCCGGCTCCAGAAT	3600	3720
62	GAGCATATTGCTTGTGGAAAGACCACCTTTTAGACTTTCAGGCCGTTCCGGCTCCAGAATCCGCTCAAAAGGGAGAACTAGCAGGACTCTTGGCGGGCTTAGCAGCCGCCCCGCCTGAAC	3660	3780
63	CCGCTCAAAAGGGAGAACTAGCAGGACTCTTGGCGGGCTTAGCAGCCGCCCCGCCTGAACCTGTAAATATATGGGTAGATTCCAAATACCTGTACTCTTTGCTCAGAACCCTAGTTCTGG	3720	3840
64	CTGTAAATATATGGGTAGATTCCAAATACCTGTACTCTTTGCTCAGAACCCTAGTTCTGGGAGCTTGGCTTCAACCTGACCCCGTACCCTCCTACGCCCTCCTATATAAAAACCTCCTCC	3780	3900
65	GAGCTTGGCTTCAACCTGACCCCGTACCCTCCTACGCCCTCCTATATAAAAACCTCCTCCGACATCCAGCAATCTTTGTTGGTCATGTCCGGAGCCACTCTTCAGCATCCCACCCTATTG	3840	3960
66	GACATCCAGCAATCTTTGTTGGTCATGTCCGGAGCCACTCTTCAGCATCCCACCCTATTGCTTCCCTGAACAATTATGTAGATCAACTGCTTCCCTTAGAAACTCCAGAGCAATGGCATA	3900	4020
67	CTTCCCTGAACAATTATGTAGATCAACTGCTTCCCTTAGAAACTCCAGAGCAATGGCATAAGCTCACCCACTGCAACTCTCGGGCCTTGTCTCGATGGCCGAACCCACGTGTCTCTGCTT	3960	4080
68	AGCTCACCCACTGCAACTCTCGGGCCTTGTCTCGATGGCCGAACCCACGTGTCTCTGCTTGGGACCCCCGTTCCCCCGCTACGCTGTGTGAAACCTGTCAAAAGCTTAATCCAACTGGAG	4020	4140
69	GGGACCCCCGTTCCCCCGCTACGCTGTGTGAAACCTGTCAAAAGCTTAATCCAACTGGAGGAGGAAGGATGCGAACTATTCAGAGAGGGTGGGCCCCGAATCATATTTGGCAGGCCGATA	4080	4200
70	GAGGAAGGATGCGAACTATTCAGAGAGGGTGGGCCCCGAATCATATTTGGCAGGCCGATATAACCCATTATAAATACAAACAGTTCACCTACGCTCTGCATGTGTTTGTAGATACTTACT	4140	4260
71	TAACCCATTATAAATACAAACAGTTCACCTACGCTCTGCATGTGTTTGTAGATACTTACTCTGGAGCTACTCATGCCTCGGCGAAGCGTGGGCTCACCACTCAAATGACTATTGAGGGCC	4200	4320
72	CTGGAGCTACTCATGCCTCGGCGAAGCGTGGGCTCACCACTCAAATGACTATTGAGGGCCTTCTTGAGGCCATAGTGCATCTGGGTCGTCCAAAAAAGCTAAACACTGACCAAGGTGCAA	4260	4380
73	TTCTTGAGGCCATAGTGCATCTGGGTCGTCCAAAAAAGCTAAACACTGACCAAGGTGCAAACTACACCTCCAAAACCTTTGTCAGGTTTTGCCAGCAGTTCGGAGTTTCCCTTTCTCATC	4320	4440
74	ACTACACCTCCAAAACCTTTGTCAGGTTTTGCCAGCAGTTCGGAGTTTCCCTTTCTCATCATGTTCCCTACAACCCCACAAGTTCGGGGTTAGTAGAACGGACAAATGGACTGCTCAAAC	4380	4500
75	ATGTTCCCTACAACCCCACAAGTTCGGGGTTAGTAGAACGGACAAATGGACTGCTCAAACTTCTTCTATCTAAATATCACCTAGACGAACCCCACCTTCCCATGACTCAGGCCCTTTCTC	4440	4560
76	TTCTTCTATCTAAATATCACCTAGACGAACCCCACCTTCCCATGACTCAGGCCCTTTCTCGAGCCCTCTGGACTCACAATCAGATTAACCTCCTACCAATTCTAAAGACCAGATGGGAGC	4500	4620
77	GAGCCCTCTGGACTCACAATCAGATTAACCTCCTACCAATTCTAAAGACCAGATGGGAGCTACACCATTCACCCCCACTTGCTGTCATTTCAGAGGGCGGAGAAACACCCAAGGGCTCTG	4560	4680
78	TACACCATTCACCCCCACTTGCTGTCATTTCAGAGGGCGGAGAAACACCCAAGGGCTCTGATAAACTCTTTTTGTACAAGCTCCCCGGGCAAAACAATCGTCGGTGGCTAGGACCACTCC	4620	4740
79	ATAAACTCTTTTTGTACAAGCTCCCCGGGCAAAACAATCGTCGGTGGCTAGGACCACTCCCGGCCCTAGTCGAAGCCTCGGGAGGCGCTCTCCTGGCTACTGACCCCCCCGTGTGGGTTC	4680	4800
80	CGGCCCTAGTCGAAGCCTCGGGAGGCGCTCTCCTGGCTACTGACCCCCCCGTGTGGGTTCCCTGGCGTTTGCTGAAAGCCTTCAAATGCCTAAAAAACGACGGTCCCGAAGACGCCCACA	4740	4860
81	CCTGGCGTTTGCTGAAAGCCTTCAAATGCCTAAAAAACGACGGTCCCGAAGACGCCCACAACCGATCATCAGATGGGTAAGTCTCACTCTCACTCTCCTCGCTCTCTGTCGGCCCATCCA	4800	4920
82	ACCGATCATCAGATGGGTAAGTCTCACTCTCACTCTCCTCGCTCTCTGTCGGCCCATCCAGACTTGGAGATGCTCCCTGTCCCTAGGAAACCAACAATGGATGACAGCATATAACCAAGA	4860	4980
83	GACTTGGAGATGCTCCCTGTCCCTAGGAAACCAACAATGGATGACAGCATATAACCAAGAGGCAAAATTTTCCATCTCCATTGACCAAATACTAGAGGCTCATAATCAGTCACCTTTCTG	4920	5040
84	GGCAAAATTTTCCATCTCCATTGACCAAATACTAGAGGCTCATAATCAGTCACCTTTCTGTGCCAAGTCTCCCAGATACACCTTGGACTCTGTAAATGGCTATCCTAAGATCTACTGGCC	4980	5100
85	TGCCAAGTCTCCCAGATACACCTTGGACTCTGTAAATGGCTATCCTAAGATCTACTGGCCCCCCCCCACAAGGGCGGCGCCGGTTTGGAGCCAGGGCCATGGTCACATATGATTGCGAGCC	5040	5160
86	CCCCCCACAAGGGCGGCGCCGGTTTGGAGCCAGGGCCATGGTCACATATGATTGCGAGCCCCGATGCCCTTATGTGGGGGCAGATCGCTTCGACTGCCCCCACTGGGACAATGCCTCCCA	5100	5220

87	CCGATGCCCTTATGTGGGGGCAGATCGCTTCGACTGCCCCCACTGGGACAATGCCTCCCAGGCCGATCAAGGATCCTTTTATGTCAATCATCAGATTTTATTCCTGCATCTTAAACAATG	5160	5280
88	GGCCGATCAAGGATCCTTTTATGTCAATCATCAGATTTTATTCCTGCATCTTAAACAATGTCATGGAATTTTCACTCTAACCTGGGAGATATGGGGATATGATCCCCTGATCACCTTTTC	5220	5340
89	TCATGGAATTTTCACTCTAACCTGGGAGATATGGGGATATGATCCCCTGATCACCTTTTCTTTACATAAGATCCCTGATCCCCCTCAACCCGACTTTCCCCAGTTGAACAGTGACTGGGT	5280	5400
90	TTTACATAAGATCCCTGATCCCCCTCAACCCGACTTTCCCCAGTTGAACAGTGACTGGGTTCCCTCTGTCAGATCATGGGCCCTGCTTTTAAACCAAACAGCACGGGCCTTCCCAGACTG	5340	5460
91	TCCCTCTGTCAGATCATGGGCCCTGCTTTTAAACCAAACAGCACGGGCCTTCCCAGACTGTGCTATATGTTGGGAACCTTCCCCTCCCTGGGCTCCCGAAATATTAGTATATAACAAAAC	5400	5520
92	TGCTATATGTTGGGAACCTTCCCCTCCCTGGGCTCCCGAAATATTAGTATATAACAAAACCATCTCCAGCTCTGGACCCGGCCTCGCCCTCCCGGACGCCCAAATCTTCTGGGTCAACAC	5460	5580
93	CATCTCCAGCTCTGGACCCGGCCTCGCCCTCCCGGACGCCCAAATCTTCTGGGTCAACACGTCCTCGTTTAACACCACCCAAGGATGGCACCACCCTTCCCAGAGGTTGTTGTTCAATGT	5520	5640
94	GTCCTCGTTTAACACCACCCAAGGATGGCACCACCCTTCCCAGAGGTTGTTGTTCAATGTTTCTCAAGGCAACGCCTTGTTATTACCTCCTATCTCCCTGGTTAATCTCTCTACGGCTTC	5580	5700
95	TTCTCAAGGCAACGCCTTGTTATTACCTCCTATCTCCCTGGTTAATCTCTCTACGGCTTCCTCCGCCCCTCCTACCCGGGTCAGACGTAGTCCCGTCGCAGCCCTGACCTTAGGCCTAGC	5640	5760
96	CTCCGCCCCTCCTACCCGGGTCAGACGTAGTCCCGTCGCAGCCCTGACCTTAGGCCTAGCCCTGTCAGTGGGGCTCACTGGAATTAATGTGGCCGTGTCTGCCCTTAGCCATCAGAGACT	5700	5820
97	CCTGTCAGTGGGGCTCACTGGAATTAATGTGGCCGTGTCTGCCCTTAGCCATCAGAGACTCACCTCCCTGATCCACGTTCTGGAGCAAGATCAGCAACGCTTGATCACAGCAATTAACCA	5760	5880
98	CACCTCCCTGATCCACGTTCTGGAGCAAGATCAGCAACGCTTGATCACAGCAATTAACCAGACCCACTATAATTTGCTTAATGTGGCCTCTGTGGTTGCCCAGAACCGACGGGGGCTTGA	5820	5940
99	GACCCACTATAATTTGCTTAATGTGGCCTCTGTGGTTGCCCAGAACCGACGGGGGCTTGATTGGTTGTACATCCGGCTGGGTTTTCAAAGCCTATGTCCCACAATTAATGAGCCTTGCTG	5880	6000
100	TTGGTTGTACATCCGGCTGGGTTTTCAAAGCCTATGTCCCACAATTAATGAGCCTTGCTGTTTCCTGCGCATTCAAAATGACTCCATTATCCGCCTCGGTGATCTCCAGCCTCTCTCGCA	5940	6060
101	TTTCCTGCGCATTCAAAATGACTCCATTATCCGCCTCGGTGATCTCCAGCCTCTCTCGCAAAGAGTCTCTACAGACTGGCAGTGGCCCTGGAATTGGGATCTGGGGCTCACTGCCTGGGT	6000	6120
102	AAGAGTCTCTACAGACTGGCAGTGGCCCTGGAATTGGGATCTGGGGCTCACTGCCTGGGTGCGAGAAACCATTCATTCTGTTCTAAGCCTGTTCCTATTAGCCCTTTTTTTGCTCTTCCT	6060	6180
103	GCGAGAAACCATTCATTCTGTTCTAAGCCTGTTCCTATTAGCCCTTTTTTTGCTCTTCCTGGCCCCCTGCCTGATAAAATGCTTGACCTCTCGCCTTTTAAAGCTCCTCCGGCAGGCTCC	6120	6240
104	GGCCCCCTGCCTGATAAAATGCTTGACCTCTCGCCTTTTAAAGCTCCTCCGGCAGGCTCCCCACTTCCCTGAAATCTCCTTAACCCCTAAACCCGATTCTGATTATCAGGCCTTGCTACC	6180	6300
105	CCACTTCCCTGAAATCTCCTTAACCCCTAAACCCGATTCTGATTATCAGGCCTTGCTACCATCTGCACCAGAGATCTACTCTCACCTCTCCCCCGTCAAACCCGATTACATCAACCTCCG	6240	6360
106	ATCTGCACCAGAGATCTACTCTCACCTCTCCCCCGTCAAACCCGATTACATCAACCTCCGACCCTGCCCTTGATACCCCCGCGTTTCACGCACCCCCAGGCTGTGGTGGTGCACTGGCTT	6300	6420
107	ACCCTGCCCTTGATACCCCCGCGTTTCACGCACCCCCAGGCTGTGGTGGTGCACTGGCTTAGTGGAATAGTCAGTGTACCATCACAAGCCTCTTCTTGCTGCCAGCACCGAGTTCGAACA	6360	6480
108	AGTGGAATAGTCAGTGTACCATCACAAGCCTCTTCTTGCTGCCAGCACCGAGTTCGAACACAGCCCTACCCTGAGCCTCTCTGAGTGCATGACTGAGTGTAGCGCAGAGAGATTGTCGCT	6420	6540
109	CAGCCCTACCCTGAGCCTCTCTGAGTGCATGACTGAGTGTAGCGCAGAGAGATTGTCGCTTCTGCGTGTCGCTCAGTCATTTTTTATAGCCGATTGGGGTTCGCGCCCTTCCGTTGCCTG	6480	6600
110	TCTGCGTGTCGCTCAGTCATTTTTTATAGCCGATTGGGGTTCGCGCCCTTCCGTTGCCTGTGACACAGATAAGACCTCTCTCACTTCTGCTTCACCATCCCCCTGCCAGCGTTGGTCTAG	6540	6660
111	TGACACAGATAAGACCTCTCTCACTTCTGCTTCACCATCCCCCTGCCAGCGTTGGTCTAGTGGAAAGAACTAACGCTGACGGGGGCGATTTCTTGCAGCTGTGCTAGCGGGAGGCTCTGG	6600	6720
112	TGGAAAGAACTAACGCTGACGGGGGCGATTTCTTGCAGCTGTGCTAGCGGGAGGCTCTGGTGCTGGGGATAAGATATGGCCCTTAGCACCATAGTCTCTGCGCCTTTTGGGTTCAAATCT	6660	6780
113	TGCTGGGGATAAGATATGGCCCTTAGCACCATAGTCTCTGCGCCTTTTGGGTTCAAATCTTCCCCACGCAGCTTCCGCTTTTTACGCCCTGTTGCACACCCTTTCTAGAGATACCTGAAA	6720	6840
114	TCCCCACGCAGCTTCCGCTTTTTACGCCCTGTTGCACACCCTTTCTAGAGATACCTGAAAATCTCAGCTCGCACCCTGAGGAGGGTTGTGGCTCAGAGGTTAAAATAGCTCGAGCCGCAA	6780	6900
115	ATCTCAGCTCGCACCCTGAGGAGGGTTGTGGCTCAGAGGTTAAAATAGCTCGAGCCGCAACCTCCCTTTCTTTTTATTCCACCCTCGCAAGGCCCCGGGTTCTGAGCCCCCTAACGGAGG	6840	6960
116	CCTCCCTTTCTTTTTATTCCACCCTCGCAAGGCCCCGGGTTCTGAGCCCCCTAACGGAGGTTCAAAATTTCCTCTACTAGGGGATGCTCGGGTCCAAGTGTGCACAATATCTCTTCCAAA	6900	7020
117	TTCAAAATTTCCTCTACTAGGGGATGCTCGGGTCCAAGTGTGCACAATATCTCTTCCAAAAGGTCCTGATGAACGTCTTCCCATGTAACAAGCCCCAGCAGAGACATTCCAGCCACATCC	6960	7080
118	AGGTCCTGATGAACGTCTTCCCATGTAACAAGCCCCAGCAGAGACATTCCAGCCACATCCAGCAGCATTTGGGCCGCCTTTTCTAACAGTGCCCATAAAGTCCCTTCCGTTTCCACAACG	7020	7140
119	AGCAGCATTTGGGCCGCCTTTTCTAACAGTGCCCATAAAGTCCCTTCCGTTTCCACAACGGCTGCCTCTGCATCTTCTATTTCCACCTCGGCACCGACTCCCCCGCCGAGCCCTTCGAGC	7080	7200
120	GCTGCCTCTGCATCTTCTATTTCCACCTCGGCACCGACTCCCCCGCCGAGCCCTTCGAGCTCTTCGGGATCCATTACCTGATAACGACAAAATTATTTCTTGTCTTTTAAGCAAGTGTTG	7140	7260
121	TCTTCGGGATCCATTACCTGATAACGACAAAATTATTTCTTGTCTTTTAAGCAAGTGTTGTTGGTTGGGGGCCCCACTCTCTACATGCCTGCCCGGCCCTGGTTTTGTCCAATGATGTCA	7200	7320
122	TTGGTTGGGGGCCCCACTCTCTACATGCCTGCCCGGCCCTGGTTTTGTCCAATGATGTCACCATCGATGCCTGGTGCCCCCTCTGCGGGCCCCATGAGCGACTCCAATTCGAAAGGATCG	7260	7380
123	CCATCGATGCCTGGTGCCCCCTCTGCGGGCCCCATGAGCGACTCCAATTCGAAAGGATCGACACCACGCTCACCTGCGAGACCCACCGTATCAACTGGACCGCCGATGGACGACCTTGCG	7320	7440
124	ACACCACGCTCACCTGCGAGACCCACCGTATCAACTGGACCGCCGATGGACGACCTTGCGGCCTCAATGGAACGTTGTTCCCTCGACTGCATGTCTCCGAGACCCGCCCCCAAGGGCCCC	7380	7500
125	GCCTCAATGGAACGTTGTTCCCTCGACTGCATGTCTCCGAGACCCGCCCCCAAGGGCCCCGACGACTCTGGATCAACTGCCCCCTTCCGGCCGTTCGCGCTCAGCCCGGCCCGGTTTCAC	7440	7560
126	GACGACTCTGGATCAACTGCCCCCTTCCGGCCGTTCGCGCTCAGCCCGGCCCGGTTTCACTTTCCCCCTTCGAGCGGTCCCCCTTCCAGCCCTACCAATGCCAATTGCCCTCGGCCTCTA	7500	7620
127	TTTCCCCCTTCGAGCGGTCCCCCTTCCAGCCCTACCAATGCCAATTGCCCTCGGCCTCTAGCGACGGTTGCCCCATTATCGGGCACGGCCTTCTTCCCTGGAACAACTTAGTAACGCATC	7560	7680
128	GCGACGGTTGCCCCATTATCGGGCACGGCCTTCTTCCCTGGAACAACTTAGTAACGCATCCTGTCCTCAGAAAAGTCCTTATATTAAATCAAATGGCCAATTTTTCCTTACTCCCCTCCT	7620	7740
129	CTGTCCTCAGAAAAGTCCTTATATTAAATCAAATGGCCAATTTTTCCTTACTCCCCTCCTTCGATACCCTCCTTGTGGACCCCCTCCGGCTGTCCGTCTTTGCCCCAGACACCAGGGGAG	7680	7800
130	TCGATACCCTCCTTGTGGACCCCCTCCGGCTGTCCGTCTTTGCCCCAGACACCAGGGGAGCCATACGTTATCTCTCCACCCTTTTGACGCTATGCCCGGCTACTTGTATTCTACCCCTAG	7740	7860
131	CCATACGTTATCTCTCCACCCTTTTGACGCTATGCCCGGCTACTTGTATTCTACCCCTAGGCGAGCCCTTCTCTCCTAATGTCCCCATATGCCGCTTTCCCCGGGACTCCAATGAACCCC	7800	7920
132	GCGAGCCCTTCTCTCCTAATGTCCCCATATGCCGCTTTCCCCGGGACTCCAATGAACCCCCCCTTTCAGAATTCGAGCTGCCCCTTATCCAAACGCCCGGCCTGTCTTGGTCTGTCCCCG	7860	7980
133	CCCTTTCAGAATTCGAGCTGCCCCTTATCCAAACGCCCGGCCTGTCTTGGTCTGTCCCCGCGATCGACCTATTCCTAACCGGTCCCCCTTCCCCATGCGACCGGTTACACGTATGGTCCA	7920	8040

134	CGATCGACCTATTCCTAACCGGTCCCCCTTCCCCATGCGACCGGTTACACGTATGGTCCAGTCCTCAGGCCTTACAGCGCTTCCTTCATGACCCTACGCTAACCTGGTCCGAATTAGTTG	7980	8100
135	GTCCTCAGGCCTTACAGCGCTTCCTTCATGACCCTACGCTAACCTGGTCCGAATTAGTTGCTAGCAGAAAAATAAGACTTGATTCCCCCTTAAAATTACAACTGCTAGAAAATGAATGGC	8040	8160
136	CTAGCAGAAAAATAAGACTTGATTCCCCCTTAAAATTACAACTGCTAGAAAATGAATGGCTCTCCCGCCTTTTTTGAGGGGGAATCATTTGTATGAAAGATCATGCCGACCTAGGCGCCG	8100	8220
137	TCTCCCGCCTTTTTTGAGGGGGAATCATTTGTATGAAAGATCATGCCGACCTAGGCGCCGCCACCGCCCCGTAAACCAGACAGAGACGTCAGCTGCCAGAAAAGCTGGTGACGGCAGCTG	8160	8280
138	CCACCGCCCCGTAAACCAGACAGAGACGTCAGCTGCCAGAAAAGCTGGTGACGGCAGCTGGTGGCTAGAATCCCCGTACCTCCCCAACTTCCCCTTTCCCGAAAAATCCACACCCTGAGC	8220	8340
139	GTGGCTAGAATCCCCGTACCTCCCCAACTTCCCCTTTCCCGAAAAATCCACACCCTGAGCTGCTGACCTCACCTGCTGATAAATTAATAAAATGCCGGCCCTGTCGAGTTAGCGGCACCA	8280	8400
140	TGCTGACCTCACCTGCTGATAAATTAATAAAATGCCGGCCCTGTCGAGTTAGCGGCACCAGAAGCGTTCTTCTCCTGAGACCCTCGTGCTCAGCTCTCGGTCCTGAGCTCTCTTGCTCCC	8340	8460
141	GAAGCGTTCTTCTCCTGAGACCCTCGTGCTCAGCTCTCGGTCCTGAGCTCTCTTGCTCCCGAGACCTTCTGGTCGGCTATCCGGCAGCGGTCAGGTAAGGCAAACCACGGTTTGGAGGGT	8400	8520
142	GAGACCTTCTGGTCGGCTATCCGGCAGCGGTCAGGTAAGGCAAACCACGGTTTGGAGGGTGGTTCTCGGCTGAGACCACCGCGAGCTCTATCTCCGGTCCTCTGACCGTCTCCACGTGGA	8460	8580
143	GGTTCTCGGCTGAGACCACCGCGAGCTCTATCTCCGGTCCTCTGACCGTCTCCACGTGGACTCTCTCCTTTGCCTCCTGACCCCGCGCTCCAAGGGCGTCTGGCTTGCACCCGCGTTTGT	8520	8640
144	CTCTCTCCTTTGCCTCCTGACCCCGCGCTCCAAGGGCGTCTGGCTTGCACCCGCGTTTGTTTCCTGTCTTACTTTCTGTTTCTCGCGGCCCGCGCTCTCTCCTTCGGCGCCCTCTAGCGG	8580	8700
145	TTCCTGTCTTACTTTCTGTTTCTCGCGGCCCGCGCTCTCTCCTTCGGCGCCCTCTAGCGGCCAGGAGAGACCGGCAAACA	8640	8720

Bos taurus TNFα promotor
(bosTau9)

1	GCCAGGGCCCCGAGAAATGGGACAACCTCCAAGGCTGGGGACTAGAGAACACCAGAGGCATTTCAGGAGACCTGGTCACACACACAGGAGCTCTCAAGGGCAGTGCTGTCTCCAGGAAAC	chr23:27537589	chr23:27537470
2	CATTTCAGGAGACCTGGTCACACACACAGGAGCTCTCAAGGGCAGTGCTGTCTCCAGGAAACTGGAGGGAGAAGGGATTCTTTGGGGATACATGGCCATACACAGGAACTCTGAAGGGGG	chr23:27537531	chr23:27537412

Table S3. List of primers used for the PCR of BLV provirus and host genome amplification

template DNA		forward primers	primer position	reverse primer	primer position (refers to EF600696)
FLK-BLV	IS1	5'-TGATGATAGCAAGGATGACAGGA-3'	chr1:245437255-245437277	5'-ATTCTACCCCTAGGCGAGCC-3'	7875-7894
	IS2	5'-ACATGGGTCAAAGTGGAAGTCTC-3'	chr2:9798865-9798887	5'-ATTCTACCCCTAGGCGAGCC-3'	7875-7894
EBL1	IS1	5'-AACAGGTCCTGAGATGATTTGCT-3'	chr14:4446983-4447005	5'-GAAACRAACGCGGGTGCAAGCCAG-3'	431-454
	IS2	5'-ACTTCCGAATGTCCACCCAC-3'	chr17:35540463-35540482	5'-GAAACRAACGCGGGTGCAAGCCAG-3'	431-454
	IS3	5'-GGCATTTTGCTGGGCACATTA-3'	chr28:41855022-41855042	5'-GAAACRAACGCGGGTGCAAGCCAG-3'	431-454
EBL2	IS1	5'-GGTCAGTCACGTCCAATGCT-3'	chr13:77065639-77065658	5'-GAAACRAACGCGGGTGCAAGCCAG-3'	431-454
	IS2	5'-TCCCTCATTTTCCCCTCAACTC-3'	chr14:70565832-70565853	5'-GTAAGGCAAACCACGGTTT-3'	306-324
EBL3	IS1	5'-CGTTGGGGACCACAAGGTAT-3'	chr1:89184077-89184096	5'-GAAACRAACGCGGGTGCAAGCCAG-3'	431-454
	IS2	5'-CGCCAATGTGTGGCTACCT-3'	chr28:15807090-15807108	5'-GTAAGGCAAACCACGGTTT-3'	306-324
EBL4	IS1	5'-ACACTGCACAACACAGAGGT-3'	chr17:51625770-51625789	5'-GAAACRAACGCGGGTGCAAGCCAG-3'	431-454

Table S4. The list of virus-host reads detected in blood samples of AS and PL cattle.

			host-left		host-right			
Sample Name	IS	Chromosome	5'end	number of reads	3'end	number of reads	Reads.total	Strand
AS	IS1	chr2	1,332,671	3	1,332,983	4	7	-
	IS2	chr3	81,083,692	4	81,084,156	3	7	+
	IS3	chr26	18,494,836	2	18,495,085	3	5	-
	IS4	chr2	22,286,843	2	22,287,220	2	4	-
	IS5	chr3	84,443,647	2	84,443,881	2	4	-
	IS6	chr2	54,227,476	2	54,227,759	1	3	-
	IS7	chr2	69,782,112	2	69,782,297	1	3	-
	IS8	chr2	89,874,882	2	89,875,103	1	3	+
	IS9	chr22	54,366,836	1	54,366,999	2	3	-
	IS10	chr24	44,276,557	1	44,276,741	2	3	+
	IS11	chr29	8,051,910	1	8,052,125	2	3	+
	IS12	chr5	88,548,310	2	88,548,480	1	3	+
	IS13	chr6	59117305	2	59117786	1	3	+
	IS14	chr7	38033612	2	38034440	1	3	-
	IS15	chr10	77499027	1	77499356	1	2	+
	IS16	chr13	36051112	1	36051486	1	2	-
	IS17	chr14	62104748	1	62104967	1	2	nd
	IS18	chr16	27383203	1	27383415	1	2	-
	IS19	chr17	3778809	1	3779043	1	2	-
	IS20	chr18	48296433	1	48296775	1	2	+
	IS21	chr19	10864368	1	10864674	1	2	+
	IS22	chr2	24557534	1	24557811	1	2	-
	IS23	chr20	13776508	1	13776921	1	2	-
	IS24	chr20	26326693	1	26327012	1	2	-
	IS25	chr23	47658635	1	47658997	1	2	-
	IS26	chr4	85547396	1	85547719	1	2	+
	IS27	chr5	26609052	1	26609190	1	2	-
	IS28	chr5	51215039	1	51215475	1	2	-
	IS29	chr7	12757203	1	12757300	1	2	+
	IS30	chr8	75538541	1	75538783	1	2	+
PL-1	IS1	chr22	7124970	8	7125342	1	9	-
	IS2	chr15	53108328	5	53108872	2	7	-
	IS3	chr9	88142752	4	88143237	2	6	+
	IS4	chr10	6938386	2	6938795	3	5	+
	IS5	chr13	61524452	1	61524753	4	5	-
	IS6	chr20	41502050	1	41502343	4	5	-
	IS7	chr21	22265694	3	22266005	2	5	+
	IS8	chr22	48136791	2	48137062	3	5	-
	IS9	chr25	36806230	3	36806705	2	5	-
	IS10	chr5	57198986	2	57199427	3	5	-
	IS11	chr1	144133349	2	144133755	2	4	-
	IS12	chr10	20752200	3	20752507	1	4	-
	IS13	chr14	354482	2	354670	2	4	+
	IS14	chr16	55167711	1	55167943	3	4	+
	IS15	chr17	14689804	3	14690108	1	4	-
	IS16	chr17	54100558	1	54100846	3	4	+
	IS17	chr18	17383939	2	17384398	2	4	+
	IS18	chr19	34084911	2	34085262	2	4	+
	IS19	chr22	50440670	1	50441070	3	4	+
	IS20	chr29	26312147	3	26312475	1	4	-
	IS21	chr29	37333928	2	37334473	2	4	-
	IS22	chr3	8346853	2	8347216	2	4	+
	IS23	chr3	50474700	1	50475150	3	4	+
	IS24	chr4	103626803	1	103627199	3	4	+
	IS25	chr9	33356673	1	33357031	3	4	+
	IS26	chr12	34113744	2	34113905	1	3	+
	IS27	chr13	12251811	2	12252108	1	3	-
	IS28	chr13	60477368	1	60477660	2	3	-
	IS29	chr13	60606672	2	60607103	1	3	+
	IS30	chr13	66219495	2	66219650	1	3	+
	IS31	chr17	62015351	1	62015518	2	3	+
	IS32	chr18	50282608	2	50283035	1	3	+
	IS33	chr19	8899932	2	8900259	1	3	-
	IS34	chr19	20808274	1	20808670	2	3	-
	IS35	chr26	4710861	2	4711055	1	3	-
	IS36	chr28	11359528	1	11359810	2	3	-
	IS37	chr28	29635304	2	29635547	1	3	+
	IS38	chr3	13355057	1	13355410	2	3	-

	IS39	chr4	46485481	2	46485992	1	3	+
	IS40	chr5	115368007	2	115368300	1	3	-
	IS41	chr6	31781455	1	31781910	2	3	-
	IS42	chr7	81110069	2	81110429	1	3	-
	IS43	chr7	100151923	2	100152244	1	3	-
	IS44	chr9	12238309	2	12238626	1	3	-
	IS45	chr1	70196461	1	70196731	1	2	+
	IS46	chr1	126704637	1	126704821	1	2	+
	IS47	chr1	128760690	1	128760925	1	2	-
	IS48	chr1	138228094	1	138228245	1	2	-
	IS49	chr10	6473900	1	6474194	1	2	+
	IS50	chr10	59780856	1	59781291	1	2	-
	IS51	chr10	64990655	1	64990846	1	2	+
	IS52	chr10	79304507	1	79304946	1	2	-
	IS53	chr11	65703887	1	65704212	1	2	+
	IS54	chr12	18221228	1	18221365	1	2	-
	IS55	chr12	28588248	1	28588357	1	2	+
	IS56	chr13	40591431	1	40591691	1	2	+
	IS57	chr13	48125441	1	48125798	1	2	+
	IS58	chr13	66134328	1	66134539	1	2	+
	IS59	chr15	2461284	1	2461871	1	2	-
	IS60	chr15	6047356	1	6047733	1	2	+
	IS61	chr15	51252828	1	51252945	1	2	+
	IS62	chr16	41676599	1	41676878	1	2	-
	IS63	chr16	51927152	1	51927417	1	2	-
	IS64	chr17	8865101	1	8865241	1	2	+
	IS65	chr17	53292971	1	53293304	1	2	-
	IS66	chr17	61529171	1	61529521	1	2	-
	IS67	chr18	20267603	1	20267759	1	2	+
	IS68	chr18	52502590	1	52502916	1	2	-
	IS69	chr2	26204736	1	26204961	1	2	+
	IS70	chr2	102530782	1	102530984	1	2	-
	IS71	chr2	119000146	1	119000410	1	2	-
	IS72	chr20	68026933	1	68027162	1	2	-
	IS73	chr21	31208863	1	31209103	1	2	-
	IS74	chr21	41290219	1	41290817	1	2	+
	IS75	chr21	45788209	1	45788578	1	2	+
	IS76	chr21	47104272	1	47104447	1	2	+
	IS77	chr22	7868620	1	7868790	1	2	-
	IS78	chr22	15891414	1	15891751	1	2	-
	IS79	chr26	5355205	1	5355697	1	2	+
	IS80	chr26	9244460	1	9244905	1	2	+
	IS81	chr26	17150524	1	17150765	1	2	-
	IS82	chr26	40647425	1	40647604	1	2	-
	IS83	chr26	42761070	1	42761228	1	2	-
	IS84	chr27	19850621	1	19850744	1	2	+
	IS85	chr28	24916070	1	24916328	1	2	+
	IS86	chr29	42301953	1	42302266	1	2	-
	IS87	chr29	43808199	1	43808384	1	2	+
	IS88	chr3	9398990	1	9399601	1	2	-
	IS89	chr3	14829164	1	14829531	1	2	+
	IS90	chr4	92796611	1	92797039	1	2	-
	IS91	chr4	119833525	1	119833749	1	2	-
	IS92	chr5	70059354	1	70059932	1	2	-
	IS93	chr5	87337159	1	87337391	1	2	+
	IS94	chr5	89537243	1	89537637	1	2	+
	IS95	chr5	97049091	1	97049300	1	2	+
	IS96	chr6	15566546	1	15566760	1	2	-
	IS97	chr6	22170164	1	22170771	1	2	+
	IS98	chr6	75080378	1	75080698	1	2	+
	IS99	chr7	18041313	1	18041606	1	2	+
	IS100	chr7	21202560	1	21202948	1	2	-
	IS101	chr8	82655671	1	82655961	1	2	+
	IS102	chr9	94441376	1	94441735	1	2	-
PL-2	IS1	chr23	2658594	76	2659376	65	141	-
	IS2	chr14	69274535	62	69275421	49	111	-
	IS3	chr21	57944280	3	57944579	5	8	-
	IS4	chr8	83912410	2	83912800	6	8	+
	IS5	chr1	35434576	4	35434960	2	6	-
	IS6	chr14	33532949	3	33533282	2	5	-
	IS7	chr5	81888805	1	81889103	4	5	+

	IS8	chr14	21066569	3	21066991	1	4	+
	IS9	chr9	24949639	1	24949859	3	4	-
	IS10	chr26	17659004	1	17659415	2	3	-
	IS11	chr26	23268342	1	23268551	2	3	+
	IS12	chr4	98103262	2	98103688	1	3	+
	IS13	chr1	58806855	1	58807028	1	2	-
	IS14	chr10	84207126	1	84207349	1	2	-
	IS15	chr13	11664470	1	11664707	1	2	-
	IS16	chr14	3738937	1	3739160	1	2	-
	IS17	chr14	16117747	1	16118060	1	2	-
	IS18	chr15	38546713	1	38546968	1	2	+
	IS19	chr15	75939373	1	75939477	1	2	-
	IS20	chr16	63676860	1	63677143	1	2	-
	IS21	chr16	65162636	1	65162728	1	2	-
	IS22	chr19	37432275	1	37432690	1	2	+
	IS23	chr2	5871539	1	5871744	1	2	-
	IS24	chr2	131840852	1	131841222	1	2	-
	IS25	chr20	63064408	1	63064713	1	2	-
	IS26	chr21	22395118	1	22395322	1	2	+
	IS27	chr24	30966014	1	30966161	1	2	+
	IS28	chr24	60446700	1	60446960	1	2	-
	IS29	chr25	1089788	1	1090090	1	2	+
	IS30	chr26	23061458	1	23061673	1	2	-
	IS31	chr28	30363242	1	30363448	1	2	+
	IS32	chr4	46502225	1	46502759	1	2	-
	IS33	chr4	100121228	1	100121641	1	2	+
	IS34	chr5	100039562	1	100039768	1	2	+
	IS35	chr5	113976877	1	113977074	1	2	+
	IS36	chr6	45850452	1	45850713	1	2	-
	IS37	chr6	57479667	1	57479920	1	2	+
	IS38	chr8	110575042	1	110575545	1	2	+
	IS39	chr9	36325857	1	36325953	1	2	-
	IS40	chr9	86492980	1	86493192	1	2	+
44	IS1	chr18	24079673	44	24080326	78	122	+
	IS2	chr15	74322841	6	74323262	6	12	-
	IS3	chr17	2683502	3	2683840	7	10	+
	IS4	chr18	36805260	6	36805600	4	10	-
	IS5	chr13	79890005	2	79890266	6	8	+
	IS6	chr19	58890294	2	58890722	6	8	+
	IS7	chr19	36213290	1	36213786	5	6	+
	IS8	chr1	492486	3	492682	2	5	+
	IS9	chr11	80941994	1	80942360	4	5	-
	IS10	chr14	42193360	4	42193730	1	5	-
	IS11	chr2	71301840	2	71302138	3	5	+
	IS12	chr23	52004609	1	52005184	4	5	-
	IS13	chr26	45579580	3	45579893	2	5	-
	IS14	chr28	13426416	1	13426699	4	5	+
	IS15	chr3	76782231	3	76782511	2	5	+
	IS16	chr5	67736579	4	67736855	1	5	+
	IS17	chr1	152432660	3	152432804	1	4	-
	IS18	chr10	26811419	2	26811766	2	4	-
	IS19	chr11	106396548	2	106396889	2	4	+
	IS20	chr2	133150930	1	133151247	3	4	+
	IS21	chr21	27267733	2	27268039	2	4	+
	IS22	chr22	5466399	2	5466714	2	4	+
	IS23	chr23	16982585	1	16982842	3	4	-
	IS24	chr25	25770384	1	25770560	3	4	+
	IS25	chr26	39834348	2	39834633	2	4	-
	IS26	chr7	45939623	1	45939956	3	4	+
	IS27	chr9	10650156	2	10650446	2	4	+
	IS28	chr10	101080602	1	101080859	2	3	+
	IS29	chr11	76286366	1	76286630	2	3	+
	IS30	chr13	67473604	1	67474090	2	3	+
	IS31	chr13	74677486	2	74678005	1	3	+
	IS32	chr16	76892001	2	76892620	1	3	+
	IS33	chr17	51221004	2	51221337	1	3	+
	IS34	chr17	54310408	1	54310708	2	3	+
	IS35	chr17	63388170	2	63388484	1	3	-
	IS36	chr25	26651647	1	26651844	2	3	+
	IS37	chr29	44088603	2	44088897	1	3	-
	IS38	chr1	38251135	1	38251393	1	2	+

IS39	chr1	44657561	1	44657987	1	2	-
IS40	chr11	16170524	1	16170691	1	2	+
IS41	chr14	59876434	1	59876695	1	2	-
IS42	chr15	63135490	1	63135650	1	2	-
IS43	chr17	53540702	1	53540992	1	2	-
IS44	chr18	46471352	1	46471534	1	2	+
IS45	chr18	53563744	1	53563990	1	2	-
IS46	chr18	62808832	1	62809090	1	2	+
IS47	chr2	116541826	1	116542012	1	2	-
IS48	chr20	28460255	1	28460512	1	2	-
IS49	chr20	71537992	1	71538174	1	2	+
IS50	chr22	16990734	1	16990845	1	2	+
IS51	chr22	50268929	1	50269216	1	2	-
IS52	chr22	58085631	1	58085887	1	2	+
IS53	chr25	14625324	1	14625551	1	2	-
IS54	chr26	21372611	1	21373247	1	2	+
IS55	chr27	20411161	1	20411487	1	2	-
IS56	chr27	25485040	1	25485385	1	2	-
IS57	chr3	2052644	1	2052793	1	2	+
IS58	chr3	107939214	1	107939484	1	2	-
IS59	chr3	108296549	1	108296923	1	2	-
IS60	chr3	110226392	1	110226517	1	2	+
IS61	chr5	7390190	1	7390603	1	2	-
IS62	chr5	74807436	1	74807741	1	2	-
IS63	chr5	76104414	1	76104717	1	2	-
IS64	chr7	15028142	1	15028383	1	2	-
IS65	chr7	49704079	1	49704281	1	2	+
IS66	chr7	69006807	1	69006978	1	2	+
IS67	chr9	67672535	1	67672774	1	2	-
IS68	chr9	70347223	1	70347537	1	2	+
IS69	chrX	85154624	1	85154841	1	2	-
IS70	chrX	102848381	1	102848772	1	2	+

IS; ID of each integration site
host-left, host-right; nucleotide position of virus-host chimeric reads as shown in Fig 2A
number of reads; the number of virus-host chimeric reads around each IS
strand; direction of BLV genome to the host genome
